# Minor Kinases with Major Roles in Cytokinesis Regulation

**DOI:** 10.3390/cells11223639

**Published:** 2022-11-17

**Authors:** Stefano Sechi, Roberto Piergentili, Maria Grazia Giansanti

**Affiliations:** Istituto di Biologia e Patologia Molecolari del Consiglio Nazionale delle Ricerche, Dipartimento di Biologia e Biotecnologie, Università Sapienza di Roma, Piazzale Aldo Moro 5, 00185 Rome, Italy

**Keywords:** cytokinesis, cancer, casein kinase 2 (CK2), P21-activated kinases (PAK), checkpoint kinase 2 (Chk2)

## Abstract

Cytokinesis, the conclusive act of cell division, allows cytoplasmic organelles and chromosomes to be faithfully partitioned between two daughter cells. In animal organisms, its accurate regulation is a fundamental task for normal development and for preventing aneuploidy. Cytokinesis failures produce genetically unstable tetraploid cells and ultimately result in chromosome instability, a hallmark of cancer cells. In animal cells, the assembly and constriction of an actomyosin ring drive cleavage furrow ingression, resulting in the formation of a cytoplasmic intercellular bridge, which is severed during abscission, the final event of cytokinesis. Kinase-mediated phosphorylation is a crucial process to orchestrate the spatio-temporal regulation of the different stages of cytokinesis. Several kinases have been described in the literature, such as cyclin-dependent kinase, polo-like kinase 1, and Aurora B, regulating both furrow ingression and/or abscission. However, others exist, with well-established roles in cell-cycle progression but whose specific role in cytokinesis has been poorly investigated, leading to considering these kinases as “minor” actors in this process. Yet, they deserve additional attention, as they might disclose unexpected routes of cell division regulation. Here, we summarize the role of multifunctional kinases in cytokinesis with a special focus on those with a still scarcely defined function during cell cleavage. Moreover, we discuss their implication in cancer.

## 1. Introduction

### 1.1. Importance of Cytokinesis in Living Cells

Cytokinesis, the conclusive process of cell division, ensures the correct distribution of cytoplasmic organelles and the nuclear content of the mother cell between two daughter cells [1]. Because of the universal requirement for cytokinesis in all dividing cells, it is not surprising that the fundamental machinery, as well as the key regulatory pathways, are conserved in animal and fungal cells [2,3]. Besides being essential for normal growth and the survival of all eukaryotic organisms, cytokinesis is also a fundamental process during animal development and for avoiding aneuploidy in adult tissues. Indeed, defects in cytokinesis have been associated with various human pathological conditions, including cancer [4,5,6]. In the context of tumorigenesis, cytokinesis failures generate genetically unstable tetraploid cells and consequently lead to chromosome instability (CIN), a hallmark of cancer cells [4,5,6,7]. Importantly, tetraploidy represents a driver event of tumorigenesis, and CIN has also been associated with cancer evolution and heterogeneity and with poor survival in cancer patients [4,5,6,7].

### 1.2. Overview of Cytokinesis in Animal Cells

**Figure 1:**
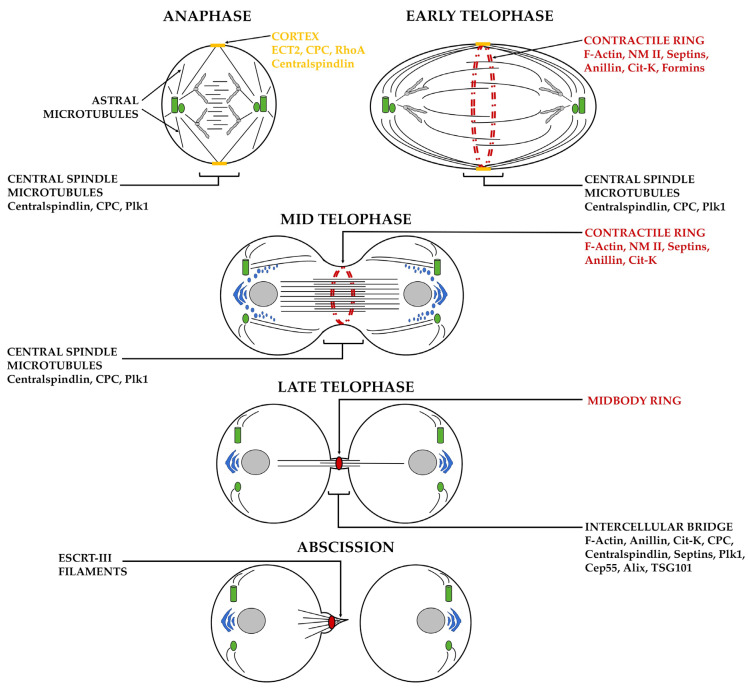
Schematic illustrating the different stages of cytokinesis in dividing animal cells and the proteins that take part in this process. Microtubules are depicted in black, centrioles in green, the contractile ring and the midbody ring in red, anaphase chromosomes and telophase nuclei in grey, Golgi organelles and Golgi-derived vesicles in blue, cortex in yellow. ECT2, epithelial cell-transforming sequence 2 oncogene; CPC, chromosomal passenger complex; RhoA, ras homolog family member A; Plk1, polo-like kinase 1; NM II, non-muscle myosin II; Cit-K, citron kinase; ESCRT-III, endosomal sorting complexes required for transport-III; Cep55, centrosomal protein 55; Alix, ALG-2-interacting protein X; TSG101, tumor susceptibility gene 101 protein.

A schematic representation of the main event occurring in animal cytokinesis is depicted in Figure 1. In animal cells, cytokinesis starts after anaphase onset and drives a profound reorganization of the spindle microtubules, which are subdivided into two distinct subpopulations, i.e., polar astral microtubules and central spindle microtubules [8]. Polar astral microtubules are dynamic structures emanating from the spindle poles and have been mainly associated with the relaxation of the non-equatorial cortex [9]. Central spindle microtubules interdigitate and overlap into antiparallel arrays at the cell equator of dividing cells [8]. Central spindle assembly and stability require the activities of many microtubule-associated proteins, kinesin-like proteins, and signaling proteins [1,8]. Signals from central spindle microtubules play an important role in specifying the Rho GTPase-dependent assembly of a contractile ring (CR), an annular structure composed of F-actin filaments and non-muscle myosin II (NM II) that is required for animal cell cytokinesis [1,2]. The evolutionarily conserved complex centralspindlin, composed of two molecules of the kinesin-6 isoform KIF23 (mitotic kinesin-like protein 1, MKLP1 in humans) and two molecules of the Rho family GTPase-activating protein (GAP) Cyk-4/MgcRacGAP, has a primary role in positioning the CR [10,11]. KIF23 enables the centralspindlin to move towards the plus ends of antiparallel microtubules, whereas Cyk-4/MgcRacGAP interacts with and recruits the Rho guanine nucleotide exchange factor ECT2 to the equatorial cortex, which activates RhoA at the cleavage site [12,13,14,15,16]. The targeting of active RhoA to the equatorial cortex activates two parallel downstream pathways, resulting in formin-dependent F-actin polymerization and NM II cortical contractility [17]. In animal cells undergoing symmetric division, the CR is assembled around the cell equator on the inner face of the plasma membrane, anchored to the plasma membrane, and connected to the central spindle by the scaffolding proteins anillin and septins [18,19,20,21]. Anillin binds to plasma membrane phosphoinositides and interacts with several proteins required for cytokinesis, such as Rho-GTP, formins, septins, F-actin, NM II, and Citron kinase/Sticky [18,19,20,21,22,23]. Septins are a family of highly conserved GTP-binding proteins that associate with each other to form filaments and higher-order oligomers at the plasma membrane [24,25,26,27]. Besides associating with anillin, septins directly bind with NM II, allowing for its full activation in dividing cells [24,25,26,27]. During the early stages of animal cell cytokinesis, actomyosin ring constriction drives cleavage furrow ingression, resulting in the formation of a cytoplasmic intercellular bridge that contains an electron-dense structure known as the midbody (MB). The MB consists of components required to regulate the final step of cytokinesis, in which the intercellular bridge is eventually severed in a process known as abscission [1,28,29,30]. At the center of the bridge, the MB forms a platform for the recruitment of proteins essential for abscission, including the endosomal sorting complex required for transport (ESCRT)-III components [28,29,30]. Abscission takes place at one side of the MB and is mediated by ESCRT-III filament helices, which are required for the final bridge constriction [31,32,33]. In human cells, abscission depends on the activity of the centrosomal-associated protein Cep55, which interacts with the centralspindlin and recruits ALIX and TSG101 proteins, which control the assembly of ESCRT-III filament helices on one side of the MB arms [33,34]. Both cleavage furrow ingression and abscission require tight crosstalk between the elements of the actin/microtubule/septin cytoskeleton and membrane lipids as well as new membrane transport from the internal secretory and endocytic/recycling trafficking compartments [1,28].

### 1.3. Spatio-Temporal Control of Cytokinesis through Phosphorylation

Reversible kinase-mediated phosphorylation contributes to a fine spatial and temporal regulation of the multiple proteins and different stages of cytokinesis [34]. Protein phosphorylation requires two main actors: protein kinases (which transfer a γ-phosphate group from ATP to serine, threonine, or tyrosine residues) and protein phosphatases (which remove it) [35,36]. Kinases and phosphatases are involved in virtually every cellular process and affect a wide range of protein behaviors and characteristics, including their activity, interactions, and localization [37,38,39]. Furthermore, the enzymatic activity of both may specifically act on only one or a few target proteins, while others are multifunctional and have a very large number of substrates [40]. The role of some multifunctional kinases in controlling crucial cytokinesis events has been extensively discussed in the literature [41,42,43,44,45,46,47]. For some of them, a clear role has been ascribed to early events of cytokinesis. For example, central spindle formation and Rho activation require the close coordination of the serine–threonine kinases polo-like mitotic kinase (Plk) 1 and Aurora B, both of which control earlier aspects of mitosis [41,42,43,44,45,46,47]. The Aurora B-mediated phosphorylation of MKLP1 controls centralspindlin formation, whereas Plk1-mediated phosphorylation of MgcRacGAP enables the centralspindlin association with ECT2 [41,42,43,44,45,46,47]. Plk1 and Aurora B also regulate the timing of abscission. The Plk1-mediated phosphorylation of Cep55 prevents its interaction with KIF23 and its accumulation at the midzone during cleavage furrow ingression [48]. In turn, by controlling the timing of Cep55 localization, Plk1 regulates the timing of ESCRT-III accumulation at the MB ring until late cytokinesis. Aurora B localizes to the MB together with the centralspindlin and phosphorylates the human ESCRT-III subunit charged multivesicular body protein 4C (CHMP4C) [49] and the abscission/NoCut checkpoint regulator (ANCHR) [50].

In this review, we will deal with those multifunctional kinases, with a well-described function in other cellular processes but whose specific role in cytokinesis is still poorly defined, making them currently “minor” actors during these stages of cell division. Indeed, recent data suggest that the number of kinases involved in cytokinesis is underestimated and that additional kinases play an important role in cell division. In particularwe report data linking three multifunctional kinases (namely, CK2, PAK, and Chk2) and different steps of cytokinesis, and how these proteins affect the central steps of this cell phase. In addition, we also report on a few papers that have shown the reciprocal crosstalk of these kinases in some aspects of cell-cycle progression, suggesting that they might act similarly during cytokinesis by interacting with each other.

## 2. Casein Kinase 2 (CK2)

### 2.1. Structure and Function of CK2

**Figure 2:**
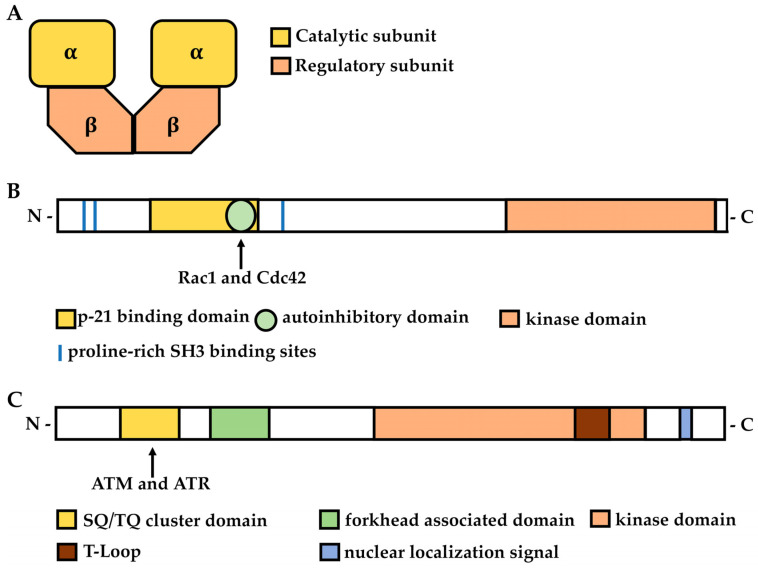
Schematic diagrams illustrating the protein structures of human CK2, PAK, and Chk2 proteins. (**A**) CK2 tetramer complex; (**B**,**C**) protein domains of PAK1 (**B**) and Chk2 proteins (**C**).

The multifunction ser/thr casein kinase 2 (CK2) is a constitutively active protein kinase that forms a hetero-tetrameric complex made of two alpha subunits with kinase activity and two regulatory beta subunits [51] (Figure 2A).

Most organisms harbor two distinct alpha subunits (alpha and alpha-prime) [52,53] and a single beta unit encoded by different genes. In contrast, the genomes of *Drosophila melanogaster* and *Schizosaccharomyces pombe* encode a single CK2 alpha and multiple beta subunit isoforms [54,55]. Mammalian CK2 complexes may contain identical (i.e., two CK2 alpha or two CK2 alpha-prime) or non-identical (i.e., one CK2 alpha and one CK2 alpha-prime) catalytic subunits [56]. CK2 kinases display messenger-independent activity, which preferentially phosphorylates ser/thr residues in the consensus sequence S/T-E/D-x-E/D [57,58] (Table 1).

CK2 localizes in both the nucleus and the cytoplasm and is also associated with specific cellular structures or organelles, including the Golgi complex, the endoplasmic reticulum (ER), and the ribosomes [73]. In addition, it has been also detected as an ecto-protein kinase at the outer surface of the plasma membrane [74,75,76,77]. This ubiquitous distribution reflects both its pleiotropic role and its involvement in many protein phosphorylation events. Indeed, ample biochemical and genetic evidence indicates the requirement for CK2 for the phosphorylation of more than 300 substrates [78]. In agreement with this, CK2 has been involved in the regulation of many cellular processes, mostly related to signaling pathways and cell-cycle control [79,80,81,82,83,84,85,86,87,88,89,90,91,92]. Although cyclin-dependent kinase 1 (CDK1), Plk1, and Aurora A/Aurora B are the most active mitotic kinases [93], multiple lines of evidence link CK2 and several aspects of the regulation of cell-cycle progression and cell division, particularly during mitotic exit [94,95,96,97,98,99,100]. For example, condensin I, a protein complex required for chromosome condensation, is inactivated by CK2 kinase-mediated phosphorylation [94]. However, during the G2/M transition, the phosphoprotein phosphatase Protein Phosphatase 6 (PP6) activates condensin I by removing this inhibitory phosphorylation [101]. Several studies on *Saccharomyces cerevisiae* have also shown that CK2 function is essential for G1/S and G2/M transitions and, indeed, CK2 depletion blocks cell-cycle progression [102,103,104,105,106]. Moreover, CK2 interacts with many proteins whose roles are required for normal progression through mitosis, such as Cdc25B, phosphoprotein phosphatase 2A (PP2A), HDAC1/2, and Topoisomerase IIα/β [107,108,109,110,111].

### 2.2. Role of CK2 in Tumorigenesis

Because CK2 is constitutively active in eukaryotic cells [112], its function in cancer development is strictly linked to its overexpression [113], which reaches particularly high levels in several types of cancer such as breast cancer, lung cancer, prostate cancer, colorectal cancer, renal cancer, various types of leukemia, and glioblastoma [114,115]. The study of CK2 action in cancerogenesis is very complex; indeed, using the PubMed search string “ck2 and cancer”, it is possible to retrieve more than 1000 results, allowing for the identification of hundreds of targets, as previously said [116,117]. This also reflects the highly dynamic behavior of CK2, which has been localized—as a response to numerous growth stimuli (such as epidermal growth factor—EGF)—to several different cellular compartments, including the Golgi apparatus, endoplasmic reticulum, mitochondria, cytoskeleton, centrosomes, and plasma membrane [118]. In addition, it has been shown that CK2 intracellular concentration increases in dividing cells and that its subcellular distribution changes from uniform in normal cells to strongly enriched in the nucleus of malignant cells [119]. The ability to interact with all DNA-dependent RNA polymerases has suggested a role for CK2 in gene regulation [120]. Among CK2 targets, we recall here the RPB1 subunit of RNA polymerase II [121], several transcription factors [122,123,124,125,126,127], various components of the spliceosome [128,129,130] and its regulators [121,131], as well as some components of the chromatin, thus influencing its remodeling (reviewed in [118]). As such, CK2 is one of the most studied kinases as a promising target for anti-cancer drug development [132]. The data in our review involve CK2 in cytokinesis, further expanding its roles in cell proliferation and possibly providing new routes of targeted therapy for cancer patients, although this role has been recently questioned [133].

### 2.3. Role of CK2 in Cytokinesis

CK2 has been implicated in the regulation of cytokinesis in several model systems. One of the first studies involving CK2 in cytokinesis dates back to the 1990s. Roussou and Draetta showed that the overexpression of Ckb1, which is the ortholog of the CK2β subunit in *S. pombe*, induces multiple septation events and impairs cell growth and cytokinesis [111]. The CK2 catalytic subunit KIN-3/CK2α of *C. elegans* was located within the nucleus, at the centrosomes, and in the MBs [112]. In nematode early embryos, the depletion of KIN-3 impairs chromosome segregation, centrosome duplication, and cytokinesis. Time-lapse imaging of worm embryos depleted of KIN-3 revealed incomplete cytokinetic furrow formation, indicating a defect in the early steps of cytokinesis [134].

Several lines of evidence have involved mammalian CK2 in late cytokinesis. The regulatory subunit CK2β and the catalytic subunit CK2α have been found localized to the MB of mammalian cells in two different studies [135,136]. A recent study on HeLa cells described a CK2-dependent regulatory mechanism linking postmitotic Golgi reassembly with the completion of cytokinesis [59]. The Golgi-specific brefeldin A-resistance guanine nucleotide exchange factor 1 (GBF1) acts as a guanine nucleotide exchange factor for the small ADP-ribosylation factor-1 (ARF1) [137,138]. In turn, the GBF1-mediated activation of ARF1 is required for maintaining Golgi architecture and intra-Golgi trafficking [138]. During mitosis, the CK2-mediated phosphorylation of GBF1 on Ser292 and Ser297 allows for its recognition by the F-box protein beta-transducin repeat containing E3 ubiquitin protein ligase (ßTrCP), which recruits GBF1 into the ubiquitin ligase complex SCFßTrCP, targeting it for degradation [59]. In turn, the phosphorylation and proteolysis of GBF1 along the microtubules of telophase cells controls Golgi inheritance and postmitotic reassembly, while the expression of a non-degradable GBF1 (S292A/S297A) mutant destabilizes the intercellular bridge and delays abscission, leading to cytokinesis failures [59]. Additionally, possible links among CK2, cytokinesis, and Golgi dynamics are suggested by the association of CK2 with NM II [60,139,140]. NM II is a collective term defining three distinct isoforms in vertebrates: non-muscle myosin IIA (NM IIA), IIB (NM IIB), and IIC (NM IIC). Our current knowledge of the function of NM II is partly derived from *Dictyostelium discoideum* and *Drosophila melanogaster.* Both model organisms harbor a single NM II encoding gene, (*mhcA* and *Zip*, respectively), facilitating genetic and biochemical analyses [141,142]. NM II mainly participates in cytokinesis [142,143] and in cell motility in conjunction with filamentous actin [144]. By using papain cleavage fragments of NM II, the 120-kDa rod domain of NM IIA heavy chain was shown to directly bind to Golgi stacks. However, this interaction was abolished when the rod domain was phosphorylated in vitro by CK2, indicating a role for CK2-mediated phosphorylation in regulating the binding and/or release of myosin-II from the Golgi [139]. Although *Drosophila* CK2 has not yet been found to be involved in cytokinesis, it has been recently found that the CK2 alpha subunit can be isolated from *Drosophila* testis extracts together with Golgi phosphoprotein 3 (GOLPH3) protein, a peripheral Golgi protein that interacts with NM II and regulates Golgi structure and actomyosin ring dynamics during cytokinesis [145]. Thus, it will be interesting to assess whether CK2 binds to and phosphorylates GOLPH3 during cytokinesis. Four phosphorylation sites on the unique C-terminus of the CK2 catalytic subunit CK2α have been involved in a regulatory mechanism controlling CK2 localization during mitosis [146]. CK2α phosphorylation controls its binding to the peptidyl-prolyl isomerase Pin1, which is required for CK2α mitotic spindle localization [146]. These data indicate the existence of a general mechanism that can direct the constitutively active CK2 kinase towards its mitotic substrates [146]. Importantly, Pin1 plays a central role during cytokinesis in regulating abscission, as shown by the analysis of Pin1 knockout in mouse embryonic fibroblasts and HeLa cells [147]. However, it remains to be investigated whether Pin1 regulates CK2 localization and activity during abscission.

In summary, research data in model organisms and human cultured cells indicate that CK2 might be involved in cytokinesis both directly, i.e., by regulating furrow ingression (in *C. elegans*), and indirectly, for example, in controlling Golgi reassembly, which is necessary for the successful completion of cytokinesis (in HeLa cells).

## 3. P21-Activated Kinase (PAK)

### 3.1. Structure and Function of PAKs

PAK serine-threonine kinases, which are conserved among eukaryotes (Figure S1), were originally identified as downstream effectors of the Rac1 and Cdc42-related GTPases [148,149]. The number of members belonging to the Pak kinase family varies among different species. *Homo sapiens* harbors six PAK members divided into two main groups: group I (PAK1 to PAK3) and group II (PAK4 to PAK6) [150,151]. *Drosophila melanogaster* has three PAK members, which are classified into group I (dPAK1, dPAK3) and group II (Mbt/dPAK2) [152]. Two PAK members have been identified in *Schizosaccharomyces pombe* (PAK1p/Orb2p/Shk1p and PAK2p/Shk2p) and three in *Saccharomyces cerevisiae*: Sterile 20 (Ste20), Cla4, and Skm1 [153,154].

PAK proteins bind to activated (GTP-bound) forms of GTPases related to Cdc42 and Rac1, but not to other small GTPases such as Ras or Rho; the result of this binding is the activation of PAKs [155,156,157]. Rac1 and Cdc42 GTPases are known to regulate F-actin, microtubules, focal adhesion assembly in cellular morphodynamics, and migration [158]. PAK family proteins usually contain an auto-inhibitory domain, a kinase domain, a p21-binding domain, and N-terminal proline-rich motifs that have the characteristic PXXP (where X indicates a variable amino acid) structure of SH3 binding domains (Figure 2B). The kinase activity of group I PAK proteins is activated after their binding to small GTPases or other proteins, whereas group II PAK members are constitutively activated [159,160,161,162]. The activity of group I PAK members is inhibited through a homodimerization mechanism. The homodimers assume a closed conformation, and the kinase activity is very low because the p21-binding domain, which overlaps with the auto-inhibitory domain, binds to the kinase domain of another PAK molecule [163,164]. The binding of Rac1 or Cdc42 proteins to the p21-binding domain of PAK1 induces a conformational change in PAK1, which leads to homodimer dissociation and an increase in its kinase activity [165]. Full kinase activity is reached after phosphorylation at Ser223 and through auto-phosphorylation at Ser144, (or at the equivalent sites for the other PAKs) [165]. The phosphorylation of PAK1 at Ser144 stabilizes the open conformation and sustains high kinase activity [166]. The regulatory mechanisms that depend on small GTPases are not the only ones that affect the activity of PAK kinases. For example, group I PAKs are stimulated by (i) the interaction of its PXXP motif with the SH3 domain of substrate molecules, e.g., Growth factor receptor-bound protein 2 (Grb2), PAK-interacting exchange (PIX), adaptor protein Nck [167,168], (ii) phosphorylation by 3-phospho-inositide dependent kinase-1, AKT and JAK [169,170], and (iii) the binding of phospholipids, exchange factor β-PIX, or SH3 proteins such as NCK1 [170] and GRB2 [167]. Instead, members of group II are constitutively active and lack the auto-inhibitory domain.

### 3.2. Role of PAKs in Tumorigenesis

The literature available in databases regarding “PAK kinase and cancer” is even more abundant than that for CK2, counting more than 1500 hits. PAK serine/threonine kinases are downstream effectors of Ras-related Rho GTPase Cdc42 and Rac [149,155,156,171], and, as such, they are involved in several oncogenic pathways, such as cell growth and proliferation, apoptosis, immune response and inflammation, motility, epithelial-to-mesenchymal transition (EMT), therapeutic resistance, angiogenesis, DNA repair, cytoskeleton remodeling, and gene expression [172]. Indeed, alterations of PAK either by mutation, upregulation, or amplification, have been linked to several cancer types with the involvement of multiple interactions with dozens of proteins, as expected from a multifunctional kinase [172]. Although some functions are shared by all six PAKs, several others are not, thus explaining the distinct types of roles in various tumors and the associated high frequency of mutation in most of them. Among PAKs, PAK1, PAK2, and PAK4 exhibit a higher expression level in most cancer types; PAK1 and PAK4 are the most studied and better characterized, yet PAK2 has the highest alteration frequency among all PAKs, especially in lung cancer [173]. In contrast, PAK6 shows both oncogenic and oncosuppressive features, depending on the cancer type. In colon and gastric cancer, the upregulation of PAK6 promotes cancer progression and chemotherapy resistance, while in clear cell renal cell carcinoma and hepatocellular carcinoma, its downregulation is linked to cancer progression and patients’ survival [174,175,176,177]. As a general rule, PAKs influence cell-cycle progression via their interaction with cyclin-dependent kinases and cyclins. Additional substrates for PAK1 include Aurora A [178], tubulin cofactor B [179], mitotic centromere-associated kinesin [180], Plk1 [181], histone H3 [182], and the epigenetic-related ATPase MORC2, a member of the Microrchidia family of proteins [183]. Several cancer-related pathways are affected by PAK malfunction; among the others, we recall here the WNT/β-catenin, EGFR/HER2/MAPK, the PI3K/AKT signaling pathways, NF-κB cascades, the EMT signaling pathway, and the DNA damage response signaling pathway [173] (and references therein). In consideration of their multiple roles, several lines of investigations have been developed over time, aimed at identifying chemical compounds impairing PAK function [184].

### 3.3. Role of PAKs in Cytokinesis

Research studies on several model organisms have revealed the role of PAK family proteins in multiple aspects related to cytokinesis (Table 1). The genome of *S. cerevisiae* encodes three members of the PAK family, of which Ste20 and Cla4 play essential yet partially redundant functions, while Skm1 is not an essential protein [185]. The inactivation of these proteins during mitosis prevents actin polarization at the bud neck, the site of constriction between the mother and the daughter cell [61]. These data indicate a role for Ste20 and Cla4 in regulating a group of polarity-determining proteins known as the polarisome, required for actin cable organization during budding yeast cytokinesis. This action could be carried out through the phosphorylation of the diaphanous-related formin Bni1p (bud neck-involved) and Bud6p [61,186,187]. It has been shown that Ste20 acts in concert with Cdc42 to control the localization of cytokinesis factors to the bud neck and allow for proper cell division [154,188]. The Cla4 kinase regulates the function of some septins during bud morphogenesis and cytokinesis [62,63,66,189,190]. In *S. cerevisiae,* the septin ring formation requires the copolymerization of the septins Cdc3p, Cdc10p, Cdc11p, Cdc12p, and Shs1/Sep7p into filaments [64,65]. Studies in vitro and in vivo have shown that Cla4 interacts directly with and phosphorylates Cdc3, Cdc10, Cdc11, and Cdc12 [62], leading to initial septin ring assembly and collar formation during bud emergence and cytokinesis [62,63,64,65,66]. Furthermore, combining Cla4 mutations with mutations affecting the septin GTP-binding domain enhances the cytokinesis defects associated with the septin collar assembly [62]. These results suggest that Cla4 organizes septins into higher-order structures necessary for cell division in a GTP-dependent manner.

In the fission yeast *S. pombe*, PAK1 is involved in cytokinesis through two mechanisms. It regulates the activation of the myosin regulatory light chain (Rlc1). Additionally, it controls the localization of several proteins necessary for the assembly of the CR, such as the anillin-related protein Mid1 and cell division control protein 15 (Cdc15) [67]. PAK1 colocalizes with the essential myosin II (Myo II) in the CR, where it phosphorylates Rlc1 at Ser35 and Ser36 [68]. In turn, the phosphorylation of Rlc1 on these residues inhibits actomyosin ring constriction and ensures the arrest of cytokinesis until complete segregation of the genetic material. The loss of PAK1 or phosphomutant forms of Rlc1 lead to premature actomyosin ring constriction [68]. PAK1 also phosphorylates the anillin-like protein Mid1 within its N-terminus and regulates its association with the plasma membrane through phosphorylation. Because contractile ring assembly and cytokinesis depend on Mid1, defects in the PAK1-Mid1 signaling pathway lead to misplaced and defective division planes [67].

Human PAK1 and PAK2 (HuPAK1 and HuPAK2) are involved in the regulation of NM II. It has been shown that the enzymatic activity of both kinases inhibits myosin light chain kinase (MLCK), which cannot activate its substrate, the myosin II regulatory light chain (MRLC) [69,191]. However, another study demonstrated that PAK directly phosphorylates MRLC and activates myosin-II [70]. These apparently conflicting observations suggest a complex regulation of myosin by PAK during cytokinesis. The HuPAK2 protein is also involved in cytokinesis. Interestingly, the tail domain of MKLP1 (amino acids 804-808,) contains a consensus sequence (K/RRXS) recognized by HuPAK2 [71,192]. Furthermore, HuPAK2 has been identified as a direct molecular interactor of MKLP1, the kinesin required for the assembly of the central spindle midzone during cytokinesis. More importantly, the MKLP1-HuPAK2 interaction is crucial for the localization of the kinesin and for the correct execution of cytokinesis [71]. Indeed, in Hek293 cells depleted of HuPAK2, MKLP1 fails to localize to the midbody, resulting in binucleated cells [71]. Taken together, these data demonstrate that protein kinases, traditionally working as mediators of actin and focal adhesion reorganization, may also carry out an important role during cytokinesis [193,194]. Interestingly, this action is also evolutionary conserved from yeast to humans, indicating that the role of PAKs in cytokinesis is likely underestimated and needs further investigation.

## 4. Checkpoint Kinase 2 (Chk2)

### 4.1. Structure and Function of Chk2

The highly conserved protein Chk2 is a serine–threonine kinase, originally discovered in *S. cerevisiae* as a Rad53-related kinase [195].

Human Chk2 protein is a single polypeptide of 543 residues containing three distinct functional domains, namely the SQ/TQ cluster domain (SCD), the forkhead associated domain (FHA), and the serine/threonine kinase domain (KD) [196] (Figure 2C). The SQ/TQ cluster domain, located at the N-terminus, is enriched in serine–glutamine and threonine–glutamine pairs [197] and contains ataxia telangiectasia-mutated (ATM) and ataxia telangiectasia and Rad3-related (ATR) phosphorylation sites [197]. The SCD domain mediates protein interactions with phosphorylated proteins, which include the phosphorylated SCD domain of a second Chk2 molecule, thus allowing for its dimerization [198]. The C-terminal half contains the kinase domain.

The activation of kinase enzymatic activity requires the phosphorylation of the Chk2 kinase domain within a region named the activation ring or T-loop, located marginally to the active site [199,200]. The T-loop contains autophosphorylated residues required for kinase activity [199,200]. The autophosphorylation of the Chk2 dimer triggers a conformational change, causing the dissociation of the dimers into fully active monomers. In addition, the C-terminal region has a nuclear localization signal [201]. An analysis of the mutations of Thr68 or changes inside the FHA domain of residues involved in phosphothreonine binding demonstrated that dimerization requires the interaction between the phosphorylated Thr68-containing SCD segment of one Chk2 molecule with the FHA domain of a second molecule [198,202]. Although Thr68 is found within an amino acid sequence recognized preferentially by kinases related to the PI3-kinase family, such as ATM, ATR, and DNA-dependent protein kinase (DNA-PK), they can also be targeted by other kinases, such as Mps1, MRK, and Plk1 [199,203,204,205]. DNA damage causes ATM kinase activation [206], which phosphorylates the Chk2 Thr68 residue, leading to Chk2 dimerization. Chk2 dimerization promotes kinase activation through autophosphorylation of the T-loop [207] (Table 1).

DNA double-strand breaks (DSBs) and related lesions arrest the cell cycle at cell-cycle checkpoints, and DNA repair is activated [208]. DNA damage response (DDR) is initiated by the activation of ATM and ATR kinases, which leads to phosphorylation and activation of Chk2. Once activated, Chk2 dissociates from the damaged sites, then amplifies and transduces the signal initiated by ATM and ATR [209,210,211].

The substrates of Chk2 include not only proteins involved in DDR, such as breast cancer type 1 susceptibility protein (BRCA1), BRCA2, and p53 [211,212,213,214], but also proteins required for cell-cycle arrest at checkpoints, such as Cdc25, whose phosphorylation by Chk2 promotes its degradation by the proteasome, thus preventing the activation of cyclin-dependent kinase 2 (Cdk2), which is necessary for the G1/S transition and progression to the S phases [210]. Moreover, in cells with irreparable damage, Chk2 also intervenes in the activation of the apoptotic pathway. In this context, a substrate of Chk2 is represented by the transcription factor E2F-1 which, once phosphorylated by the kinase, promotes the transcription of proapoptotic genes [215].

### 4.2. Role of Chk2 in Tumorigenesis

A PubMed search of “Chk2 and cancer” had plenty of results, with the total number of retrieved articles exceeding 1500. As said, this serine–threonine kinase is a cell-cycle checkpoint regulator mainly involved in cell-cycle arrest during G1 and S in response to DNA damage [210,216]. Chk2 was first identified in 1998 [195], and it was shown that its conserved, single FHA domain is essential for its ATM-dependent activation in response to stimuli [216]. Chk2 is activated through phosphorylation in response to ionizing radiation and hydroxyurea treatment, although a functional ATM is required only for the first type of DNA insult [217]. In turn, Chk2 regulates multiple targets through phosphorylation; beyond the above-mentioned BRCA1 [218], E2F-1 [215], and Cdc25 [210], its interactors also include the NBS1/MRE11 axis [219], the inducer of acute promyelocytic leukemia, PML, [220], and the ATR/ATM/p53 pathway, both of which induce apoptosis [221]. The role of CK2 as an oncosuppressor was first hypothesized in 1999, when Bell and coworkers found frequent heterozygous mutations in Li–Fraumeni syndrome patients, who are ca. 25x more cancer-prone than average [222]. These data were subsequently confirmed by other groups [223,224], yet the final role of Chk2 in this syndrome is still a debated topic [225]. Nonetheless, evidence has accumulated over the years for the involvement of CHK2 in cancer, with reports of CHK2 mutations also found in sarcoma, breast cancer, colon cancer, ovarian cancer, osteosarcoma, lung cancer, prostate cancer, kidney cancer, thyroid cancer, and brain tumors [222,224,226,227,228,229,230]. Some reports also associated CHK2 with mitotic spindle damage, through its action on BRCA1 [231]; these data, coupled with those linking this kinase to cytokinesis, indicate CHK2 as a possible target for treating cancer cells using drugs aimed at impairing the last phases of cell division [232]. 

### 4.3. Role of Chk2 in Cytokinesis

The G1/S transition is not the only checkpoint activated by Chk2. Indeed, a recent study on cultured human cells involved human Chk2 in the activation of the abscission checkpoint [72]. The abscission checkpoint, which delays cytokinesis in response to chromosome segregation defects [233,234,235] to prevent chromatin breakage and tetraploidization by regression of the cleavage furrow, is maintained by Aurora B kinase localized to the MB [236,237]. The activity and recruitment of Aurora B are strictly dependent on its partners in the chromosomal passenger complex (CPC), which includes the scaffolding protein INCENP and the subunits Survivin and Borealin [47,238]. In turn, the localization of the CPC requires the binding of INCENP to Mklp2 kinesin [239,240]. It was demonstrated that ATM phosphorylates and activates Chk2 at the MB center in late cytokinesis in normal cells and that active Chk2 phosphorylates human INCENP on Ser91 to promote INCENP binding to Mklp2 [72]. In turn, INCENP allows Aurora B localization to the MB, which delays abscission in normally segregating cells [72]. If these data were confirmed, then the localization and activation of Aurora B at abscission would be significantly dependent on Chk2 function, thus opening new routes for understanding the events leading to the final separation of daughter cells in eukaryotes. This would add a new, important player in the field of cytokinesis completion.

## 5. On the Crosstalk between Minor Kinases—Novel Insight into Cell-Cycle Control?

It is noteworthy to underline that a few reports have highlighted functional links among the three kinases described here, suggesting that, at least in some instances, they cooperate in regulating the cell cycle. It has been shown that CK2α colocalizes with PAK1 through the association with CKIP-1 at the plasma membrane as a response to EGF, and CK2α phosphorylates PAK1, promoting its cancer-related functions [165,241]. It has been demonstrated by Zhang and collaborators that the overexpression of PAK5 can downregulate p-CHK2 in hepatocellular carcinoma cells [242]. Kim showed that CK2 interacts with Chk2 and phosphorylates MRE11, thus indicating CK2 as a potential upstream regulator of MRE11 function [243]. Bjørling-Poulsen and coworkers showed that the beta-subunit of CK2 specifically interacts with Chk2 in vitro and in cultured cells, and that the activation of Chk2 leads to a reduction of this interaction. Moreover, the presence of the CK2 beta-subunit significantly reduced the Chk2-catalyzed phosphorylation of p53 in vitro [244]. Finally, Kroonen and collaborators showed that CK2 protein depletion inhibited NF-κB activation and altered the Tyr68 phosphorylation of Chk2 in malignant glioma cells [245]. Although these data need further validation, the crosslinks among these three kinases unveil a novel network of cell-cycle controllers that surely needs additional investigation. The advances in linking these three kinases to cytokinesis either directly or indirectly further confirm their role in tumorigenesis, increasing the complexity of their interactome but also expanding the possible routes of investigation regarding possible therapeutic approaches in cancer treatment. In this context, recent work has shown that inducing cytokinesis failures could provide an effective strategy to block cell division and promote cell death in cancer cells [246,247,248].

## 6. Conclusions

The events occurring during cytokinesis are under strict genetic control. The complexity of these events is reflected by the number of proteins involved in the completion of cell division. Some of these proteins have a structural role, being an integral part of molecular architectures that build up structures such as MB or CR (myosin, kinesin, etc.). Others have a gene regulation function and are responsible for determining which proteins should be present in a given cell phase (e.g., E2F-1). Finally, a third group of proteins controls the function of other target proteins by post-translational modifications, mainly phosphorylation, such as kinases. Their action is exerted in several different ways; they can activate or deactivate the substrate protein or promote its degradation via the proteasome pathway. Many kinases have been strongly linked to cytokinesis, their role is well known, and most of their targets, as well as the effects of their mutation, are described in the literature; examples include cyclin-dependent kinase, polo-like, and Aurora B. However, the number of kinases involved in cytokinesis is likely underestimated. Increasing evidence is accumulating, describing the role of multifunctional kinases in this process. To date, the data available about these enzymes are still fragmented and need both further validation and additional investigation. Yet, their role in cell division is far from unnecessary or merely redundant. Rather, their role can be central in this process, as their targets in some cases include the well-known above-mentioned kinases; for example, the action of Chk2 on Aurora B. Understanding the role of these multifunctional kinases may allow for identifying novel ways of controlling the final phases of cell division, a phenomenon that is at the basis of neoplastic transformation. Indeed, the connection between cytokinesis failure and tumorigenesis is a well-established fact [249]. Moreover, cancer treatment can be accomplished not only by trying to patch cytokinesis impairment but also by inducing it [247]. Thus, identifying the main actors involved in cytokinesis and learning how they interact and how to alter their function (either inhibiting or activating them) is crucial to discover new, useful approaches for the development of new diagnostic and/or therapeutic strategies in cancer treatment. Indeed, protein kinases are one of the most successful drug targets for oncology due to their critical role in different mechanisms that drive malignant transformation [21,22,23], and discovering additional functions in the cytokinesis of undervalued kinases will be pivotal for improving the research in this field.

## Figures and Tables

**Table 1:** Table illustrating the roles of the CK2, Pak, and Chk2 kinases in cytokinesis, identified in model organisms and/or human cultured cells, and the relative phosphorylation target proteins. GBF1, Golgi brefeldin A-resistant guanine nucleotide exchange factor 1; NM II, non-muscle myosin II; NMHC II, non-muscle myosin II heavy chain; Bni1, diaphanous-related formin; Mid1, anillin-related medial ring protein; Rlc1, non-muscle myosin II regulatory light chain; Cdc15, cell division control protein 15; MLCK, non-muscle myosin II light chain kinase; MRLC, non-muscle myosin II regulatory light chain; MKLP1, mitotic kinesin-like protein1; CPC, chromosomal passenger complex.

CK2 Kinase	Organism	Cytokinesis Functions	Phosphorylation Target	Phosphorylated Residues	Ref.
CK2	*Homo sapiens*	Intercellular bridge stabilization	GBF1	Ser292 Ser297	[59]
CK2	*Homo sapiens*	NM II filament disassembly	NMHC II	Ser1944	[60]
**PAK** **Kinase**	**Organism**	**Cytokinesis** **Functions**	**Phosphorylation** **Target**	**Phosphorylated** **Residues**	**Ref.**
Ste20	*Saccharomyces* *cerevisiae*	Formin activation, F-actin ring assembly and dynamics	Bni1	ND	[61]
Cla4	*Saccharomyces* *cerevisiae*	Formin activation, F-actin ring assembly and dynamics	Bni1	ND	[61]
Cla4	*Saccharomyces* *cerevisiae*	Septin ring assembly	Septins	ND	[62,63,64,65,66]
Pak1	*Schizosaccharomyces pombe*	CR positioning	Mid1	N-terminus phosphorylation	[67]
Pak1	*Schizosaccharomyces pombe*	CR assembly	Cdc15	ND	[67]
Pak1	*Schizosaccharomyces pombe*	CR constriction	Rlc1	Ser35 and Ser36	[68]
Pak2	*Homo sapiens*	MLCK inhibition	MLCK	Ser439 and Ser991	[69]
Pak2	*Homo sapiens*	MRLC activation	MRLC	Ser19	[70]
Pak2	*Homo sapiens*	Midbody dynamics	MKLP1	tail domain *	[71]
**Chk2** **Kinase**	**Organism**	**Cytokinesis** **Functions**	**Phosphorylation** **Target**	**Phosphorylated** **Residues**	**Ref.**
Chk2	*Homo sapiens*	CPC localization	INCENP	Ser91	[72]

* PAK2 consensus sequence (K/RRXS) in the tail domain, amino acids 804–808.

## Supplementary Materials

The following supporting information can be downloaded at: https://www.mdpi.com/article/10.3390/cells11223639/s1, Figure S1: Percent identity of the kinase domain of the PAKs mentioned in the text. The reference sequences used were identified with the Prosite software and the alignment was done with Clustal Omega.

## Abbreviations

**Acronyms**	**Full name**
ALIX	ALG-2-interacting protein X
AKT	Protein kinase B
ANCHR	Abscission/NoCut checkpoint regulator
ARF1	ADP-ribosylation factor-1
ATM	Ataxia telangiectasia-mutated
ATR	Ataxia telangiectasia and Rad3-related
Bni1	Diaphanous-related formin
ßPIX	PAK-interacting exchange factor beta
BRCA1	Breast cancer type 1 susceptibility protein
BRCA2	Breast cancer type 2 susceptibility protein
ßTrCP	Beta-transducin repeat containing E3 ubiquitin protein ligase
Bud6	Bud site selection protein 6
Cdc3	Cell division control protein 3
Cdc10	Cell division control protein 10
Cdc11	Cell division control protein 11
Cdc12	Cell division control protein 12
Cdc15	Cell division control protein 15
Cdc25	Cell division cycle 25
Cdc25B	Cell division cycle 25B
Cdc42	Cell division control protein 42 homolog
CDK1	Cyclin-dependent kinase 1
Cep55	Centrosomal protein 55
Chk2	Checkpoint kinase 2
CHMP4C	Charged multivesicular body protein 4C
CIN	Chromosome instability
Cit-K	Citron kinase
CK2	Casein kinase 2
CK2α	Casein kinase 2 subunit alpha
CK2β	Casein kinase 2 subunit beta
Ckb1	Casein kinase 2 subunit beta
CKIP-1	Casein kinase 2 interacting protein 1
Cla4	Serine/threonine protein kinase CLA4
CPC	Chromosomal passenger complex
CR	Contractile ring
Cyk-4	Cytokinesis defect-4
DNA-PK	DNA-dependent protein kinase
DDR	DNA damage response
E2F1	Transcription factor
ECT2	Epithelial cell-transforming sequence 2 oncogene
EGF	Epidermal growth factor
EGFR	Epidermal growth factor receptor
ER	Endoplasmic reticulum
ESCRT-III	Endosomal sorting complexes required for transport-III
FHA	Forkhead-associated domain
GBF1	Golgi brefeldin A-resistant guanine nucleotide exchange factor 1
GOLPH3	Golgi phosphoprotein 3
GRB2	Growth factor receptor-bound protein 2
HDAC1	Histone deacetylase 1
HDAC2	Histone deacetylase 2
INCENP	Inner centromere protein
JAK	Janus tyrosine kinase
KD	Serine/threonine kinase domain
KIF23	Kinesin-like protein
MAPK	Mitogen-activated protein Kinases
MB	Midbody
mbt	Mushroom bodies tiny
MgcRacGAP	Rac GTPase-activating protein 1
Mid1	Anillin-related medial ring protein mid1
mhcA	*Myosin heavy chain A*
MKLP1	Mitotic kinesin-like protein1
MLCK	Myosin light chain kinase
MORC2	Microrchidia family CW-type zinc finger protein 2
MPS1	MonoPolar spindle 1
MRE11	Double-strand break repair protein MRE11
MRK	MLK-related kinase
MRLC	Myosin II regulatory light chain
Myo II	Myosin II
NF-kB	Nuclear factor kappa-light-chain-enhancer of activated B cells
NBS1	Nijmegen breakage syndrome 1
Nck	Non-catalytic region of tyrosine kinase
NM IIA	Non-muscle myosin II isoform A
Orb2p	Translational regulator orb2
PAK	P21-activated kinases
PI3K	Phosphatidylnositol 3-kinase
PIN1	Peptidyl-prolyl cis-trans isomerase NIMA-interacting 1
PIX	PAK-interacting exchange
Plk1	Polo-like kinase 1
PML	Promyelocytic leukemia
PP2A	Phosphoprotein phosphatase 2A
PP6	Protein phosphatase 6
PXXP	Proline-Xaa-Xaa-Proline
Rac1	Ras-related C3 botulinum toxin substrate 1
Rlc1	Myosin regulatory light chain
RhoA	Ras homolog family member A
SCD	SQ/TQ cluster domain
SCF	Skp1/Cul-1/F-box protein complex
Sep7	Septation protein 7
SH3	SRC homology 3 domain
Shs1	Seventh homolog of septin 1
Shk1	Serine/threonine protein kinase shk1/pak1
Shk2	Serine/threonine protein kinase shk2
Skm1	Serine/threonine protein kinase shk1/pak1
Ste20	Serine/threonine protein kinase STE20
TSG101	Tumor susceptibility gene 101 protein
*zip*	*Zipper*

## References

[1] D’Avino P.P., Giansanti M.G., Petronczki M. (2015). Cytokinesis in animal cells. Cold Spring Harb. Perspect. Biol..

[2] Pollard T.D., O’Shaughnessy B. (2020). Molecular Mechanism of Cytokinesis. Annu. Rev. Biochem..

[3] Bhavsar-Jog Y.P., Bi E. (2017). Mechanics and regulation of cytokinesis in budding yeast. Semin. Cell Dev. Biol..

[4] Wangsa D., Quintanilla I., Torabi K., Vila-Casadesús M., Ercilla A., Klus G., Yuce Z., Galofré C., Cuatrecasas M., Lozano J.J. (2018). Near-tetraploid cancer cells show chromosome instability triggered by replication stress and exhibit enhanced invasiveness. FASEB J..

[5] Bach D.H., Zhang W., Sood A.K. (2019). Chromosomal Instability in Tumor Initiation and Development. Cancer Res..

[6] Telentschak S., Soliwoda M., Nohroudi K., Addicks K., Klinz F.J. (2015). Cytokinesis failure and successful multipolar mitoses drive aneuploidy in glioblastoma cells. Oncol. Rep..

[7] Heng H.H., Bremer S.W., Stevens J.B., Horne S.D., Liu G., Abdallah B.Y., Ye K.J., Ye C.Y. (2013). Chromosomal instability (CIN): What it is and why it is crucial to cancer evolution. Cancer Metastasis Rev..

[8] Mishima M. (2016). centralsplindin in Rappaport’s cleavage signaling. Semin. Cell Dev. Biol..

[9] Murthy K., Wadsworth P. (2008). Dual role for microtubules in regulating cortical contractility during cytokinesis. J. Cell Sci..

[10] Mishima M., Kaitna S., Glotzer M. (2002). Central spindle assembly and cytokinesis require a kinesin-like protein/ RhoGAP complex with microtubule bundling activity. Dev. Cell.

[11] White-Cooper H., Caporilli S. (2013). Transcriptional and post-transcriptional regulation of Drosophila germline stem cells and their differentiating progeny. Adv. Exp. Med. Biol..

[12] Somers W.G., Saint R. (2003). A RhoGEF and Rho family GTPase-activating protein complex links the contractile ring to cortical microtubules at the onset of cytokinesis. Dev. Cell.

[13] Yuce O., Piekny A., Glotzer M. (2005). An ECT2-centralspindlin complex regulates the localization and function of RhoA. J. Cell Biol..

[14] Zhao W.M., Fang G. (2005). MgcRacGAP controls the assembly of the contractile ring and the initiation of cytokinesis. Proc. Natl. Acad. Sci. USA.

[15] Kamijo K., Ohara N., Abe M., Uchimura T., Hosoya H., Lee J.S., Miki T. (2006). Dissecting the role of Rho-mediated signaling in contractile ring formation. Mol. Biol. Cell.

[16] Nishimura Y., Yonemura S. (2006). Centralspindlin regulates ECT2 and RhoA accumulation at the equatorial cortex during cytokinesis. J. Cell Sci..

[17] Piekny A., Werner M., Glotzer M. (2005). Cytokinesis: Welcome to the Rho zone. Trends Cell Biol..

[18] Kim O.V., Litvinov R.I., Mordakhanova E.R., Bi E., Vagin O., Weisel J.W. (2022). Contribution of septins to human platelet structure and function. iScience.

[19] Garno C., Irons Z.H., Gamache C.M., McKim Q., Reyes G., Wu X., Shuster C.B., Henson J.H. (2021). Building the cytokinetic contractile ring in an early embryo: Initiation as clusters of myosin II, anillin and septin, and visualization of a septin filament network. PLoS ONE.

[20] Kučera O., Siahaan V., Janda D., Dijkstra S.H., Pilátová E., Zatecka E., Diez S., Braun M., Lansky Z. (2021). Anillin propels myosin-independent constriction of actin rings. Nat. Commun..

[21] Carim S.C., Kechad A., Hickson G.R.X. (2020). Animal Cell Cytokinesis: The Rho-dependent Actomyosin-Anillo septin Contractile Ring as a Membrane Microdomain Gathering, Compressing, and Sorting Machine. Front. Cell Dev. Biol..

[22] Gai M., Camera P., Dema A., Bianchi F., Berto G., Scarpa E., Germena G., Di Cunto F. (2011). Citron kinase controls abscission through RhoA and anillin. Mol. Biol. Cell.

[23] El-Amine N., Carim S.C., Wernike D., Hickson G.R.X. (2019). Rho-dependent control of the Citron kinase. Sticky, drives midbody ring maturation. Mol. Biol. Cell.

[24] Sirajuddin M., Farkasovsky M., Hauer F., Kühlmann D., Macara I.G., Weyand M., Stark H., Wittinghofer A. (2007). Structural insight into filament formation by mammalian septins. Nature.

[25] Piatti S. (2020). Cytokinesis: An Anillin-RhoGEF Module Sets the Stage for Septin Double Ring Assembly. Curr. Biol..

[26] Kinoshita M., Field C.M., Coughlin M.L., Straight A.F., Mitchison T.J. (2002). Self- and Actin-Templated Assembly of Mammalian Septins. Dev. Cell.

[27] Joo E., Surka M.C., Trimble W.S. (2007). Mammalian SEPT2 Is Required for Scaffolding Nonmuscle Myosin II and its Kinases. Dev. Cell.

[28] Fremont S., Echard A. (2018). Membrane traffic in the late steps of cytokinesis. Curr. Biol..

[29] Hu C.K., Coughlin M., Mitchison T.J. (2012). Midbody assembly and its regulation during cytokinesis. Mol. Biol. Cell.

[30] Petsalaki E., Zachos G. (2021). The Abscission Ceckpoint: A guardian of chromosomal stability. Cells.

[31] Carlton J.Z., Martin-Serrano J. (2007). Parallels between cytokinesis and retroviral budding: A role for the ESCRT machinery. Science.

[32] Horváth P., Müller-Reichert T. (2020). A Structural View on ESCRT-Mediated Abscission. Front. Cell Dev. Biol..

[33] Morita E., Sandrin V., Chung H.Y., Morham S.G., Gygi S.P., Rodesch C.K., Sundquist W.I. (2007). Human ESCRT and ALIX proteins interact with proteins of the midbody and function in cytokinesis. EMBO J..

[34] Lee H.H., Elia N., Ghirlando R., Lippincott-Schwartz J., Hurley J.H. (2008). Midbody targeting of the ESCRT machinery by a noncanonical coiled coil in CEP55. Science.

[35] Nasa I., Kettenbach A.N. (2018). Coordination of Protein Kinase and Phosphoprotein Phosphatase Activities in Mitosis. Front. Cell Dev. Biol..

[36] Smoly I., Shemesh N., Ziv-Ukelson M., Ben-Zvi A., Yeger-Lotem E. (2017). An Asymmetrically Balanced Organization of Kinases versus Phosphatases across Eukaryotes Determines Their Distinct Impacts. PLoS Comput. Biol..

[37] Manning G., Plowman G.D., Hunter T., Sudarsanam S. (2002). Evolution of protein kinase signaling from yeast to man. Trends Biochem. Sci..

[38] Bononi A., Agnoletto C., De Marchi E., Marchi S., Patergnani S., Bonora M., Giorgi C., Missiroli S., Poletti F., Rimessi A. (2011). Protein kinases and phosphatases in the control of cell fate. Enzym. Res..

[39] Audagnotto M., Dal Peraro M. (2017). Protein post-translational modifications: In silico prediction tools and molecular modeling. Comput. Struct. Biotechnol. J..

[40] Pinna L.A., Ruzzene M. (1996). How do protein kinases recognize their substrates?. Biochim. Et. Biophys. Acta.

[41] Brennan I.M., Peters U., Kapoor T.M., Straight A.F. (2007). Polo-like kinase controls vertebrate spindle elongation and cytokinesis. PLoS ONE.

[42] Santamaria A., Neef R., Eberspächer U., Eis K., Husemann M., Mumberg D., Prechtl S., Schulze V., Siemeister G., Wortmann L. (2007). Use of the novel Plk1 inhibitor ZK-thiazolidinone to elucidate functions of Plk1 in early and late stages of mitosis. Mol. Biol. Cell.

[43] Burkard M.E., Maciejowski J., Rodriguez-Bravo V., Repka M., Lowery D.M., Clauser K.R., Zhang C., Shokat K.M., Carr S.A., Yaffe M.B. (2009). Plk1 self-organization and priming phosphorylation of HsCYK-4 at the spindle midzone regulate the onset of division in human cells. PLoS Biol..

[44] Wolfe B.A., Takaki T., Petronczki M., Glotzer M. (2009). Polo-like kinase 1 directs assembly of the HsCyk-4 RhoGAP/Ect2 RhoGEF complex to initiate cleavage furrow formation. PLoS Biol..

[45] Barr F.A., Silljé H.H., Nigg E.A. (2004). Polo-like kinases and the orchestration of cell division. Nat. Rev. Mol. Cell Biol..

[46] Van der Waal M.S., Hengeveld R.C., Van der Horst A., Lens S.M. (2012). Cell division control by the Chromosomal Passenger Complex. Exp. Cell Res..

[47] Carmena M., Wheelock M., Funabiki H., Earnshaw W.C. (2012). The chromosomal passenger complex (CPC): From easy rider to the godfather of mitosis. Nat. Rev. Mol. Cell Biol..

[48] Bastos R.N., Barr F.A. (2010). Plk1 negatively regulates Cep55 recruitment to the midbody to ensure orderly abscission. J. Cell Biol..

[49] Capalbo L., Montembault E., Takeda T., Bassi Z.I., Glover D.M., D’Avino P.P. (2012). The chromosomal passenger complex controls the function of endosomal sorting complex required for transport-III Snf7 proteins during cytokinesis. Open Biol..

[50] Thoresen S.B., Campsteijn C., Vietri M., Schink K.O., Liestøl K., Andersen J.S., Raiborg C., Stenmark H. (2014). ANCHR mediates Aurora-B-dependent abscission checkpoint control through retention of VPS4. Nat. Cell Biol..

[51] Glover C.V.C., Shelton E.R., Brutlag D.L. (1983). Purification and characterization of a type II casein kinase from Drosophila melanogaster. J. Biol. Chem..

[52] Dahmus M.E. (1981). Purification and properties of calf thymus casein kinase I and II. J. Biol. Chem..

[53] Litchfield D.W., Lozeman F.J., Piening C., Sommercorn J., Takio K., Walsh K.A., Krebs E.G. (1990). Subunit structure of casein kinase II from bovine testis. Demonstration that the α and α′ subunits are distinct polypeptides. J. Biol. Chem..

[54] Hathaway G.M., Zoller M.J., Traugh J.A. (1981). Identification of the catalytic subunit of casein kinase II by affinity labeling with 5′-p-fluorosulfonylbenzoyl adenosine. J. Biol. Chem..

[55] Meggio F., Brunati A.M., Pinna L.A. (1983). Autophosphorylation of type 2 casein kinase TS at both its α- and β- subunits. FEBS Lett..

[56] Gietz R.D., Graham K.C., Litchfield D.W. (1995). Interactions between the subunits of casein kinase II. J. Biol. Chem..

[57] Kuenzel E.A., Mulligan J.A., Sommercorn J., Krebs E.G. (1987). Substrate specificity determinants for casein kinase II as deduced from studies with synthetic peptides. J. Biol. Chem..

[58] Meggio F., Marin O., Pinna L.A. (1994). Substrate specificity of protein kinase CK2. Cell. Mol. Biol. Res..

[59] Magliozzi R., Carrero Z.I., Low T.Y., Yuniati L., Valdes-Quezada C., Kruiswijk F., Van Wijk K., Heck A.J.R., Jackson C.L., Guardavaccaro D. (2018). Inheritance of the Golgi Apparatus and Cytokinesis Are Controlled by Degradation of GBF1. Cell Rep..

[60] Dulyaninova N.G., Malashkevich V.N., Almo S.C., Bresnick A.R. (2005). Regulation of Myosin-IIA Assembly and Mts1 Binding by Heavy Chain Phosphorylation. Biochemistry.

[61] Goehring A.S., Mitchell D.A., Tong A.H., Keniry M.E., Boone C., Sprague G.F. (2003). Synthetic lethal analysis implicates Ste20p, a p21-activated protein kinase, in polarisome activation. Mol. Biol. Cell.

[62] Versele M., Thorner J. (2004). Septin collar formation in budding yeast requires GTP binding and direct phosphorylation by the PAK, Cla4. J. Cell Biol..

[63] Weiss E.L., Bishop A.C., Shokat K.M., Drubin D.G. (2000). Chemical genetic analysis of the budding-yeast p21-activated kinase Cla4p. Nat. Cell Biol..

[64] Frazier J.A., Wong M.L., Longtine M.S., Pringle J.R., Mann M., Mitchison T.J., Field C. (1998). Polymerization of purified yeast septins: Evidence that organized filament arrays may not be required for septin function. J. Cell Biol..

[65] Field C.M., Kellogg D. (1999). Septins: Cytoskeletal polymers or signalling GTPases?. Trends Cell Biol..

[66] Kadota J., Yamamoto T., Yoshiuchi S., Bi E., Tanaka K. (2004). Septin ring assembly requires concerted action of polarisome components, a PAK kinase Cla4p, and the actin cytoskeleton in Saccharomyces cerevisiae. Mol. Biol. Cell.

[67] Magliozzi J.O., Sears J., Cressey L., Brady M., Opalko H.E., Kettenbach A.N., Moseley J.B. (2020). Fission yeast Pak1 phosphorylates anillin-like Mid1 for spatial control of cytokinesis. J. Cell Biol..

[68] Loo T.H., Balasubramanian M. (2008). Schizosaccharomyces pombe Pak-related protein, Pak1p/Orb2p, phosphorylates myosin regulatory light chain to inhibit cytokinesis. J. Cell Biol..

[69] Goeckeler Z.M., Masaracchia R.A., Zeng Q., Chew T.L., Gallagher P., Wysolmerski R.B. (2000). Phosphorylation of Myosin Light Chain Kinase by p21-activated Kinase PAK2. J. Biol. Chem..

[70] Chew T.L., Masaracchia R.A., Goeckeler Z.M., Wysolmerski R.B. (1998). Phosphorylation of non-muscle myosin II regulatory light chain by p21-activated kinase (gamma-PAK). J. Muscle Res. Cell Motil..

[71] Zhang Z.H., Liu X.L., Zhu Y.Y., Huang H. (2020). Revealing PAK2′s Function in the Cell Division through MKLP1′s Interactome. Biomed Res. Int..

[72] Petsalaki E., Zachos G. (2020). An ATM–Chk2–INCENP pathway activates the abscission checkpoint. J. Cell Biol..

[73] Litchfield D.W. (2003). Protein kinase CK2: Structure, regulation and role in cellular decisions of life and death. Biochem. J..

[74] Faust M., Montenarh M. (2000). Subcellular localization of protein kinase CK2. A key to its function?. Cell Tissue Res..

[75] Sarrouilhe D., Filhol O., Leroy D., Bonello G., Baudry M., Chambaz E., Cochet C. (1998). The tight association of protein kinase CK2 with plasma membrane is mediated by a specific domain of its regulatory β-subunit. Biochim. Biophys. Acta.

[76] Dittie A.S., Thomas L., Thomas G., Tooze S.A. (1997). Interaction of furin in immature secretory granules from neuroendocrine cells with the AP-1 adaptor complex is modulated by casein kinase II phosphorylation. EMBO J..

[77] Walter J., Schnolzer M., Pyerin W., Kinzel V., Kubler D. (1996). Induced release of cell surface protein kinase yields CK1- and CK2-like enzymes in tandem. J. Biol. Chem..

[78] Meggio F., Pinna L.A. (2003). One-thousand-and-one substrates of protein kinase CK2?. FASEB J..

[79] Montenarh M. (2016). Protein kinase CK2 in DNA damage and repair. Transl. Cancer Res..

[80] Pinna L., Meggio F. (1997). Protein kinase CK2 (‘‘casein kinase-2′’) and its implication in cell division and proliferation. Prog. Cell Cycle Res..

[81] Ahmed K. (1999). Nuclear matrix and protein kinase CK2 signaling. Crit. Rev. Eukaryot. Gene Expr..

[82] Guerra B., Issinger O.G. (1999). Protein kinase CK2 and its role in cellular proliferation, development and pathology. Electrophoresis.

[83] Ahmed K., Gerber D.A., Cochet C. (2002). Joining the cell survival squad: An emerging role for protein kinase CK2. Trends Cell Biol..

[84] Fragoso R., Barata J.T. (2015). Kinases, tails and more: Regulation of PTEN function by phosphorylation. Methods.

[85] Ruzzene M., Bertacchini J., Toker A., Marmiroli S. (2017). Cross-talk between the CK2 and AKT signaling pathways in cancer. Adv. Biol. Regul..

[86] Dominguez I., Sonenshein G.E., Seldin D.C. (2009). Protein kinase CK2 in health and disease: CK2 and its role in Wnt and NF-kappaB signaling: Linking development and cancer. Cell. Mol. Life Sci..

[87] Manni S., Brancalion A., Mandato E., Quotti Tubi L., Colpo A., Pizzi M., Cappellesso R., Zaffino F., Di Maggio S.A., Cabrelle A. (2013). Protein kinase CK2 inhibition down modulates the NF-κB and STAT3 survival pathways, enhances the cellular proteotoxic stress and synergistically boosts the cytotoxic effect of bortezomib on multiple myeloma and mantle cell lymphoma cells. PLoS ONE.

[88] Zheng Y., Qin H., Frank S.J., Deng L., Litchfield D.W., Tefferi A., Pardanani A., Lin F.T., Li J., Sha B. (2011). A CK2-dependent mechanism for activation of the JAK-STAT signaling pathway. Blood.

[89] Jia H., Liu Y., Xia R., Tong C., Yue T., Jiang J., Jia J. (2010). Casein kinase 2 promotes Hedgehog signaling by regulating both smoothened and Cubitus interruptus. J. Biol. Chem..

[90] Litchfield D.W., Lüscher B. (1993). Casein kinase II in signal transduction and cell cycle regulation. Mol. Cell. Biochem..

[91] Zhang S., Long H., Yang Y.L., Wang Y., Hsieh D., Li W., Au A., Stoppler H.J., Xu Z., Jablons D.M. (2013). Inhibition of CK2α down-regulates Notch1 signalling in lung cancer cells. J. Cell. Mol. Med..

[92] De Gooijer M.C., Guillén N.M., Bernards R., Wurdinger T., Van Tellingen O. (2018). An experimenter’s guide to glioblastoma invasion pathways. Trends Mol. Med..

[93] Salaun P., Rannou Y., Prigent C. (2008). Cdk1, Plks, Auroras, and Neks: The mitotic bodyguards. Adv. Exp. Med. Biol..

[94] Takemoto A., Kimura K., Yanagisawa J., Yokoyama S., Hanaoka F. (2006). Negative regulation of condensin I by CK2-mediated phosphorylation. EMBO J..

[95] Yde C.W., Olsen B.B., Meek D., Watanabe N., Guerra B. (2008). The regulatory beta-subunit of protein kinase CK2 regulates cell-cycle progression at the onset of mitosis. Oncogene.

[96] St-Denis N.A., Derksen D.R., Litchfield D.W. (2009). Evidence for regulation of mitotic progression through temporal phosphorylation and dephosphorylation of CK2α. Mol. Cell. Biol..

[97] St-Denis N., Gabriel M., Turowec J.P., Gloor G.B., Li S.S., Gingras A.C., Litchfield D.W. (2015). Systematic investigation of hierarchical phosphorylation by protein kinase CK2. J. Proteom..

[98] Li H., Liu X.S., Yang X., Wang Y., Wang Y., Turner J.R., Liu X. (2010). Phosphorylation of CLIP-170 by Plk1 and CK2 promotes timely formation of kinetochore-microtubule attachments. EMBO J..

[99] Barrett R.M., Colnaghi R., Wheatley S.P. (2011). Threonine 48 in the BIR domain of survivin is critical to its mitotic and anti-apoptotic activities and can be phosphorylated by CK2 in vitro. Cell Cycle.

[100] Peng Y., Wong C.C., Nakajima Y., Tyers R.G., Sarkeshik A.S., Yates J., Drubina D.G., Barnes G. (2011). Overlapping kinetochore targets of CK2 and Aurora B kinases in mitotic regulation. Mol. Biol. Cell.

[101] Rusin S.F., Schlosser K.A., Adamo M.E., Kettenbach A.N. (2015). Quantitative phosphoproteomics reveals new roles for the protein phosphatase PP6 in mitotic cells. Sci. Signal..

[102] Pepperkok R., Lorenz P., Jakobi R., Ansorge W., Pyerin W. (1991). Cell growth stimulation by EGF: Inhibition through antisense- oligodeoxynucleotides demonstrates important role of casein kinase II. Exp. Cell Res..

[103] Pepperkok R., Lorenz P., Ansorge W., Pyerin W. (1994). Casein kinase II is required for transition of G0/G1, early G1, and G1/S phases of the cell cycle. J. Biol. Chem..

[104] Lorenz P., Pepperkok R., Ansorge W., Pyerin W. (1993). Cell biological studies with monoclonal and polyclonal antibodies against human casein kinase II subunit beta demonstrate participation of the kinase in mitogenic signaling. J. Biol. Chem..

[105] Glover C.V. (1998). On the physiological role of casein kinase II in Saccharomyces cerevisiae. Prog. Nucleic Acid Res. Mol. Biol..

[106] Hériché J.K., Lebrin F., Rabilloud T., Leroy D., Chambaz E.M., Goldberg Y. (1997). Regulation of protein phosphatase 2A by direct interaction with casein kinase 2α. Science.

[107] Daum J.R., Gorbsky G.J. (1998). Casein kinase II catalyzes a mitotic phosphorylation on threonine 1342 of human DNA topoisomerase IIalpha, which is recognized by the 3F3/2 phosphoepitope antibody. J. Biol. Chem..

[108] Escargueil A.E., Plisov S.Y., Filhol O., Cochet C., Larsen A.K. (2000). Mitotic phosphorylation of DNA topoisomerase II alpha by protein kinase CK2 creates the MPM-2 phosphoepitope on Ser-1469. J. Biol. Chem..

[109] Theis-Febvre N., Filhol O., Froment C., Cazales M., Cochet C., Monsarrat B., Ducommun B., Baldin B. (2003). Protein kinase CK2 regulates CDC25B phosphatase activity. Oncogene.

[110] Khan D.H., He S., Yu J., Winter S., Cao W., Seiser C., Davie J.R. (2013). Protein kinase CK2 regulates the dimerization of histone deacetylase 1 (HDAC1) and HDAC2 during mitosis. J. Biol. Chem..

[111] Roussou I., Draetta G. (1994). The Schizosaccharomyces pombe casein kinase II alpha and beta subunits: Evolutionary conservation and positive role of the beta subunit. Mol. Cell. Biol..

[112] Singh N.N., Ramji D.P. (2008). Protein kinase CK2, an important regulator of the inflammatory response?. J. Mol. Med..

[113] Ortega C.E., Seidner Y., Dominguez I. (2014). Mining CK2 in cancer. PLoS ONE.

[114] Iegre J., Atkinson E.L., Brear P.D., Cooper B.M., Hyvönen M., Spring D.R. (2021). Chemical probes targeting the kinase CK2: A journey outside the catalytic box. Org. Biomol. Chem..

[115] Pucko E.B., Ostrowski R.P. (2022). Inhibiting CK2 among Promising Therapeutic Strategies for Gliomas and Several Other Neoplasms. Pharmaceutics.

[116] Pinna L.A. (2002). Protein kinase CK2: A challenge to canons. J. Cell Sci..

[117] Rusin S.F., Adamo M.E., Kettenbach A.N. (2017). Identification of Candidate Casein Kinase 2 Substrates in Mitosis by Quantitative Phosphoproteomics. Front. Cell Dev. Biol..

[118] Trembley J.H., Kren B.T., Afzal M., Scaria G.A., Klein M.A., Ahmed K. (2022). Protein kinase CK2-diverse roles in cancer cell biology and therapeutic promise. Mol. Cell. Biochem..

[119] Faust R.A., Niehans G., Gapany M., Hoistad D., Knapp D., Cherwitz D., Davis A., Adams G.L., Ahmed K. (1999). Subcellular immunolocalization of protein kinase CK2 in normal and carcinoma cells. Int. J. Biochem. Cell Biol..

[120] Nozawa R.S., Gilbert N. (2019). RNA: Nuclear Glue for Folding the Genome. Trends Cell Biol..

[121] Trembley J.H., Hu D., Slaughter C.A., Lahti J.M., Kidd V.J. (2003). Casein kinase 2 interacts with cyclin-dependent kinase 11 (CDK11) in vivo and phosphorylates both the RNA polymerase II carboxyl-terminal domain and CDK11 in vitro. J. Biol. Chem..

[122] Cabrejos M.E., Allende C.C., Maldonado E. (2004). Effects of phosphorylation by protein kinase CK2 on the human basal components of the RNA polymerase II transcription machinery. J. Cell. Biochem..

[123] Palancade B., Dubois M.F., Bensaude O. (2002). FCP1 phosphorylation by casein kinase 2 enhances binding to TFIIF and RNA polymerase II carboxyl-terminal domain phosphatase activity. J. Biol. Chem..

[124] Újvári A., Pal M., Luse D.S. (2011). The functions of TFIIF during initiation and transcript elongation are differentially affected by phosphorylation by casein kinase 2. J. Biol. Chem..

[125] Yamaguchi Y., Wada T., Suzuki F., Takagi T., Hasegawa J., Handa H. (1998). Casein kinase II interacts with the bZIP domains of several transcription factors. Nucleic Acids Res..

[126] Bird T.A., Schooley K., Dower S.K., Hagen H., Virca G.D. (1997). Activation of Nuclear Transcription Factor NF-κB by Interleukin-1 Is Accompanied by Casein Kinase II-mediated Phosphorylation of the p65 Subunit. J. Biol. Chem..

[127] Voit R., Schnapp A., Kuhn A., Rosenbauer H., Hirschmann P., Stunnenberg H.G., Grummt I. (1992). The nucleolar transcription factor mUBF is phosphorylated by casein kinase II in the C-terminal hyperacidic tail which is essential for transactivation. EMBO J..

[128] Bian Y., Ye M., Wang C., Cheng K., Song C., Dong M., Pan Y., Qin H., Zou H. (2013). Global screening of CK2 kinase substrates by an integrated phosphoproteomics workflow. Sci. Rep..

[129] Trembley J.H., Tatsumi S., Sakashita E., Loyer P., Slaughter C.A., Suzuki H., Endo H., Kidd V.J., Mayeda A. (2005). Activation of pre-mRNA splicing by human RNPS1 is regulated by CK2 phosphorylation. Mol. Cell. Biol..

[130] Lehnert S., Gotz C., Kartarius S., Schafer B., Montenarh M. (2008). Protein kinase CK2 interacts with the splicing factor hPrp3p. Oncogene.

[131] Mylonis I., Giannakouros T. (2003). Protein kinase CK2 phosphorylates and activates the SR protein-specific kinase 1. Biochem. Biophys. Res. Commun..

[132] Borgo C., D’Amore C., Sarno S., Salvi M., Ruzzene M. (2021). Protein kinase CK2: A potential therapeutic target for diverse human diseases. Signal Transduct. Target. Ther..

[133] Salvi M., Borgo C., Pinna L.A., Ruzzene M. (2021). Targeting CK2 in cancer: A valuable strategy or a waste of time?. Cell Death Discov..

[134] Medley J.C., Kabara M.M., Stubenvoll M.D., DeMeyer L.E., Song M.H. (2017). Casein kinase II is required for proper cell division and acts as a negative regulator of centrosome duplication in Caenorhabditis elegans embryos. Biol. Open..

[135] Skop A.R., Liu H., Yates J., Meyer B.J., Heald R. (2004). Dissection of the mammalian midbody proteome reveals conserved cytokinesis mechanisms. Science.

[136] Salvi M., Raiborg C., Hanson P.I., Campsteijn C., Stenmark H., Pinna L.A. (2014). CK2 involvement in ESCRT-III complex phosphorylation. Arch. Biochem. Biophys..

[137] Donaldson J.G., Jackson C.L. (2000). Regulators and effectors of the ARF GTPases. Curr. Opin. Cell Biol..

[138] Donaldson J.G., Jackson C.L. (2011). ARF family G proteins and their regulators: Roles in membrane transport, development and disease. Nat. Rev. Mol. Cell Biol..

[139] Fath K.R. (2005). Characterization of myosin-II binding to Golgi stacks in vitro. Cell Motil. Cytoskelet..

[140] Manstein D.J., Titus M.A., De Lozanne A., Spudich J.A. (1989). Gene replacement in Dictyostelium: Generation of myosin null mutants. EMBO J..

[141] Young P.E., Richman A.M., Ketchum A.S., Kiehart D.P. (1993). Morphogenesis in Drosophila requires nonmuscle myosin heavy chain function. Genes Dev..

[142] Sechi S., Frappaolo A., Karimpour-Ghahnavieh A., Fraschini R., Giansanti M.G. (2020). A novel coordinated function of Myosin II with GOLPH3 controls centralspindlin localization during cytokinesis in Drosophila. J. Cell Sci..

[143] Babkoff A., Cohen-Kfir E., Aharon H., Ravid S. (2021). Aurora-B phosphorylates the myosin II heavy chain to promote cytokinesis. J. Biol. Chem..

[144] Betapudi V., Gokulrangan G., Chance M.R., Egelhoff T.T. (2011). A proteomic study of myosin II motor proteins during tumor cell migration. J. Mol. Biol..

[145] Sechi S., Karimpour-Ghahnavieh A., Frappaolo A., Di Francesco L., Piergentili R., Schininà E., D’Avino P.P., Giansanti M.G. (2021). Identification of GOLPH3 Partners in Drosophila Unveils Potential Novel Roles in Tumorigenesis and Neural Disorders. Cells.

[146] St-Denis N.A., Bailey M.L., Parker E.L., Vilk G., Litchfield D.W. (2011). Localization of phosphorylated CK2alpha to the mitotic spindle requires the peptidyl-prolyl isomerase Pin1. J. Cell Sci..

[147] Van der Horst A., Khanna K.K. (2009). The peptidyl-prolyl isomerase Pin1 regulates cytokinesis through Cep55. Cancer Res..

[148] Manser E., Leung T., Salihuddin H., Zhao Z.S., Lim L. (1994). A brain serine/threonine protein kinase activated by Cdc42 and Rac1. Nature.

[149] Knaus U.G., Morris S., Dong H.J., Chernoff J., Bokoch G.M. (1995). Regulation of human leukocyte p21-activated kinases through G protein–coupled receptors. Science.

[150] Rane C.K., Minden A. (2019). P21 activated kinase signaling in cancer. Semin. Cancer Biol..

[151] Hofmann C., Shepelev M., Chernoff J. (2004). The genetics of Pak. J. Cell Sci..

[152] Mentzel B., Raabe T. (2005). Phylogenetic and structural analysis of the Drosophila melanogaster p21-activated kinase DmPAK3. Gene.

[153] Lin M., Unden H., Jacquier N., Schneiter R., Just U., Höfken T. (2009). The Cdc42 effectors Ste20, Cla4, and Skm1 down-regulate the expression of genes involved in sterol uptake by a mitogen-activated protein kinase-independent pathway. Mol. Biol. Cell.

[154] Qyang Y., Yang P., Du H., Lai H., Kim H., Marcus S. (2002). The p21-activated kinase, Shk1, is required for proper regulation of microtubule dynamics in the fission yeast, Schizosaccharomyces pombe. Mol. Microbiol..

[155] Martin G.A., Bollag G., McCormick F., Abo A. (1995). A novel serine kinase activated by rac1/CDC42Hs-dependent autophosphorylation is related to PAK65 and STE20. EMBO J..

[156] Bagrodia S., Taylor S.J., Creasy C.L., Chernoff J., Cerione R.A. (1995). Identification of a mouse p21Cdc42/Rac activated kinase. J. Biol. Chem..

[157] Sells M.A., Barratt J.T., Caviston J., Ottilie S., Leberer E., Chernoff J. (1998). Characterization of Pak2p, a pleckstrin homology domain-containing, p21-activated protein kinase from fission yeast. J. Biol. Chem..

[158] Iden S., Collard J.G. (2008). Crosstalk between small GTPases and polarity proteins in cell polarization. Nat. Rev. Mol. Cell Biol..

[159] Rane C.K., Minden A. (2014). P21 activated kinases: Structure, regulation, and functions. Small GTPases.

[160] Jha R.K., Strauss C.E.M. (2012). 3D structure analysis of PAKs: A clue to the rational design for affinity reagents and blockers. Cell. Logist..

[161] Baker N.M., Chow H.Y., Chernoff J., Der C.J. (2014). Molecular pathways: Targeting RAC-p21-activated serine-threonine kinase signaling in RAS-driven cancers. Clin. Cancer Res..

[162] Pirruccello M., Sondermann H., Pelton J.G., Pellicena P., Hoelz A., Chernoff J., Wemmer D.E., Kuriyan J. (2006). A dimeric kinase assembly underlying autophosphorylation in the p21 activated kinases. J. Mol. Biol..

[163] Buchwald G., Hostinova E., Rudolph M.G., Kraemer A., Sickmann A., Meyer H.E., Scheffzek K., Wittinghofer A. (2001). Conformational switch and role of phosphorylation in PAK activation. Mol. Cell. Biol..

[164] Parrini M.C. (2012). Untangling the complexity of PAK1 dynamics: The future challenge. Cell. Logist..

[165] Shin Y.J., Kim Y.B., Kim J.H. (2013). Protein kinase CK2 phosphorylates and activates p21-activated kinase 1. Mol. Biol. Cell.

[166] Chong C., Tan L., Lim L., Manser E. (2001). The mechanism of PAK activation. Autophosphorylation events in both regulatory and kinase domains control activity. J. Biol. Chem..

[167] Puto L.A., Pestonjamasp K., King C.C., Bokoch G.M. (2003). p21-activated kinase 1 (PAK1) interacts with the Grb2 adapter protein to couple to growth factor signaling. J. Biol. Chem..

[168] Zhao Z.S., Manser E., Lim L. (2000). Interaction between PAK and Nck: A Template for Nck Targets and Role of PAK Autophosphorylation. Mol. Cell. Biol..

[169] Zhou G.L., Zhuo Y., King C.C., Fryer B.H., Bokoch G.M., Field J. (2003). Akt phosphorylation of serine 21 on Pak1 modulates Nck binding and cell migration. Mol. Cell. Biol..

[170] Fryer B.H., Wang C., Vedantam S., Zhou G.L., Jin S., Fletcher L., Simon M.C., Fiel J. (2006). cGMP-dependent protein kinase phosphorylates p21-activated kinase (Pak) 1, inhibiting Pak/Nck binding and stimulating Pak/vasodilator-stimulated phosphoprotein association. J. Biol. Chem..

[171] Morrice N.A., Gabrielli B., Kemp B.E., Wettenhall R.E.A. (1994). Cardiolipin-activated protein kinase from rat liver structurally distinct from the protein kinases C. J. Biol. Chem..

[172] Kumar R., Li D.Q. (2016). PAKs in human cancer progression: From inception to cancer therapeutic to future oncobiology. Adv. Cancer Res..

[173] Liu H., Liu K., Dong Z. (2021). The Role of p21-Activated Kinases in Cancer and Beyond: Where Are We Heading?. Front. Cell Dev. Biol..

[174] Liu W., Liu H., Liu Y., Xu L., Zhang W., Zhu Y., Xu J., Gu J. (2014). Prognostic significance of p21-activated kinase 6 expression in patients with clear cell renal cell carcinoma. Ann. Surg. Oncol..

[175] Liu W., Liu Y., Liu H., Zhang W., Fu Q., Xu J. (2015). Tumor suppressive function of p21-activated Kinase 6 in hepatocellular carcinoma. J. Biol. Chem..

[176] Chen J., Lu H., Yan D., Cui F., Wang X., Yu F., Xue Y., Feng X., Wang J., Wang X. (2015). PAK6 increase chemoresistance and is a prognostic marker for stage II and III colon cancer patients undergoing 5-FU based chemotherapy. Oncotarget.

[177] Jiang Y., Liu W., Li T., Hu Y., Chen S., Xi S., Wen Y., Huang L., Zhao L., Xiao C. (2017). Prognostic and predictive value of p21-activated Kinase 6 associated support vector machine classifier in gastric cancer treated by 5-fluorouracil/oxaliplatin chemotherapy. EBioMedicine.

[178] Zhao Z.S., Lim J.P., Ng Y.W., Lim L., Manser E. (2005). The GIT-associated kinase PAK targets to the centrosome and regulates Aurora-A. Mol. Cell..

[179] Vadlamudi R.K., Barnes C.J., Rayala S., Li F., Balasenthil S., Marcus S. (2005). p21-activated kinase 1 regulates microtubule dynamics by phosphorylating tubulin cofactor B. Mol. Cell. Biol..

[180] Pakala S.B., Nair V.S., Reddy S.D., Kumar R. (2012). Signaling-dependent phosphorylation of mitotic centromere-associated kinesin regulates microtubule depolymerization and its centrosomal localization. J. Biol. Chem..

[181] Maroto B., Ye M.B., von Lohneysen K., Schnelzer A., Knaus U.G. (2008). P21-activated kinase is required for mitotic progression and regulates Plk1. Oncogene.

[182] Li F., Adam L., Vadlamudi R.K., Zhou H., Sen S., Chernoff J., Mandal M., Kumar R. (2002). p21-activated kinase 1 interacts with and phosphorylates histone H3 in breast cancer cells. EMBO Rep..

[183] Li D.Q., Nair S.S., Ohshiro K., Kumar A., Nair V.S., Pakala S.B., Reddy S.D.N., Gajula R.P., Eswaran J., Aravind L. (2012). MORC2 signaling integrates phosphorylation-dependent, ATPase-coupled chromatin remodeling during the DNA damage response. Cell Rep..

[184] Rudolph J., Crawford J.J., Hoeflich K.P., Wang W. (2015). Inhibitors of p21-Activated Kinases (PAKs). J. Med. Chem..

[185] Juanes M.A., Piatti S. (2016). The final cut: Cell polarity meets cytokinesis at the bud neck in S. cerevisiae. Cell Mol. Life Sci..

[186] Cvrckova F., De Virgilio C., Manser E., Pringle J.R., Nasmyth K. (1995). Ste20-like protein kinases are required for normal localization of cell growth and for cytokinesis in budding yeast. Genes Dev..

[187] Annan R.B., Lee A.Y., Reid I.D., Sayad A., Whiteway M., Hallett M., Thomas D.Y. (2009). A biochemical genomics screen for substrates of Ste20p kinase enables the in silico prediction of novel substrates. PLoS ONE.

[188] Atkins B.D., Yoshida S., Saito K., Wu C.F., Lew D.J., Pellman D. (2013). Inhibition of Cdc42 during mitotic exit is required for cytokinesis. J. Cell Biol..

[189] Dobbelaere J., Gentry M.S., Hallberg R.L., Barral Y. (2003). Phosphorylation-dependent regulation of septin dynamics during the cell cycle. Dev. Cell.

[190] Schmidt M., Varma A., Drgon T., Bowers B., Cabib E. (2003). Septins, under Cla4p regulation, and the chitin ring are required for neck integrity in budding yeast. Mol. Biol. Cell.

[191] Sanders L.C., Matsumura F., Bokoch G.M., de Lanerolle P. (1999). Inhibition of myosin light chain kinase by p21-activated kinase. Science.

[192] Tuazon P.T., Spanos W.C., Gump E.L., Monnig C.A., Traugh J.A. (1997). Determinants for substrate phosphorylation by p21-activated protein kinase (gamma-PAK). Biochemistry.

[193] Sells M.A., Knaus U.G., Bagrodia S., Ambrose D.M., Bokoch G.M., Chernoff J. (1997). Human p21-activated kinase (Pak1) regulates actin organization in mammalian cells. Curr. Biol..

[194] Frost J.A., Khokhlatchev A., Stippec S., White M.A., Cobb M.H. (1998). Differential effects of Pak1-activating mutations reveal activity-dependent and -independent effects on cytoskeletal regulation. J. Biol. Chem..

[195] Matsuoka S., Huang M., Elledge S.J. (1998). Linkage of ATM to cell cycle regulation by the Chk2 protein kinase. Science.

[196] Cai Z., Chehab N.H., Pavletich N.P. (2009). Structure and Activation Mechanism of the CHK2 DNA Damage Checkpoint Kinase. Mol. Cell.

[197] Buscemi G., Carlessi L., Zannini L., Lisanti S., Fontanella E., Canevari S., Delia D. (2006). DNA damage-induced cell cycle regulation and function of novel Chk2 phosphoresidues. Mol. Cell. Biol..

[198] Li J., Williams B.L., Haire L.F., Goldberg M., Wilker E., Durocher D., Yaffe M.B., Jackson S.P., Smerdon S.J. (2002). Structural and functional versatility of the FHA domain in DNA-damage signaling by the tumor suppressor kinase Chk2. Mol. Cell.

[199] Zannini L., Delia D., Buscemi G. (2014). CHK2 kinase in the DNA damage response and beyond. J. Mol. Cell Biol..

[200] Schwarz J.K., Lovly C.M., Piwnica-Worms H. (2003). Regulation of the Chk2 protein kinase by oligomerization-mediated cis- and trans-phosphorylation. Mol. Cancer Res..

[201] Zannini L., Lecis D., Lisanti S., Benetti R., Buscemi G., Schneider C., Delia D. (2003). Karyopherin-alpha2 protein interacts with Chk2 and contributes to its nuclear import. J. Biol. Chem..

[202] Xu X., Tsvetkov L.M., Stern D.F. (2002). Chk2 activation and phosphorylation-dependent oligomerization. Mol. Cell. Biol..

[203] Tosti E., Waldbaum L., Warshaw G., Gross E.A., Ruggieri R. (2004). The stress kinase MRK contributes to regulation of DNA damage checkpoints through a p38g-independent pathway. J. Biol. Chem..

[204] Wei J.H., Chou Y.F., Ou Y.H., Yeh Y.H., Tyan S.W., Sun T.P., Shen C.Y., Shieh S.Y. (2005). TTK/hMps1 Participates in the Regulation of DNA Damage Checkpoint Response by Phosphorylating CHK2 on Threonine 68. J. Biol. Chem..

[205] Tsvetkov L., Xu X., Li J., Stern D.F. (2003). Polo-like kinase 1 and Chk2 interact and co-localize to centrosomes and the midbody. J. Biol. Chem..

[206] Ahn J.Y., Schwarz J.K., Piwnica-Worms H., Canman C.E. (2000). Threonine 68 phosphorylation by ataxia telangiectasia mutated is required for efficient activation of Chk2 in response to ionizing radiation. Cancer Res..

[207] Matsuoka S., Rotman G., Ogawa A., Shiloh Y., Tamai K., Elledge S.J. (2000). Ataxia telangiectasia- mutated phosphorylates Chk2 in vivo and in vitro. Proc. Natl. Acad. Sci. USA.

[208] Zhou B.B., Elledge S.J. (2000). The DNA damage response: Putting checkpoints in perspective. Nature.

[209] Abraham R.T. (2001). Cell cycle checkpoints: Preventing an identity crisis. Genes Dev..

[210] Falck J., Mailand N., Syljuasen R.G., Bartek J., Lukas J. (2001). The ATM-Chk2-Cdc25A checkpoint pathway guards against radioresistant DNA synthesis. Nature.

[211] Ahn J., Urist M., Prives C. (2004). The Chk2 protein kinase. DNA Repair (Amst.).

[212] Matsuoka S., Ballif B.A., Smogorzewska A., McDonald E.R., Hurov K.E., Luo J., Bakalarski C.E., Zhao Z., Solimini N., Lerenthal Y. (2007). ATM and ATR substrate analysis reveals extensive protein networks responsive to DNA damage. Science.

[213] Gatei M., Scott S.P., Filippovitch I., Soronika N., Lavin M.F., Weber B., Khannaet K.K. (2000). Role for ATM in DNA damage-induced phosphorylation of BRCA1. Cancer Res..

[214] Banin S., Moyal L., Shieh S., Taya Y., Anderson C.W., Chessa L., Smorodinsky N.I., Prives C., Reiss Y., Shiloh Y. (1998). Enhanced phosphorylation of p53 by ATM in response to DNA damage. Science.

[215] Stevens C., Smith L., La Thangue N.B. (2003). Chk2 activates E2F-1 in response to DNA damage. Nat. Cell Biol..

[216] Chehab N.H., Malikzay A., Appel M., Halazonetis T.D. (2000). Chk2/hCds1 functions as a DNA damage checkpoint in G-1 by stabilizing p53. Genes Dev..

[217] Brown A.L., Lee C.H., Schwarz J.K., Mitiku N., Piwnica-Worms H., Chung J.H. (1999). A human Cds1-related kinase that functions downstream of ATM protein in the cellular response to DNA damage. Proc. Natl. Acad. Sci. USA.

[218] Lee J.S., Collins K.M., Brown A.L., Lee C.H., Chung J.H. (2000). hCds1-mediated phosphorylation of BRCA1 regulates the DNA damage response. Nature.

[219] Falck J., Petrini J.H.J., Williams B.R., Lukas J., Bartek J. (2002). The DNA damage-dependent intra-S phase checkpoint is regulated by parallel pathways. Nat. Genet..

[220] Yang S., Kuo C., Bisi J.E., Kim M.K. (2002). PML-dependent apoptosis after DNA damage is regulated by the checkpoint kinase hCds1/Chk2. Nat. Cell Biol..

[221] Bartkova J., Horejsi Z., Koed K., Kramer A., Tort F., Zieger K., Guldberg P., Sehested M., Nesland J.M., Lukas C. (2005). DNA damage response as a candidate anti-cancer barrier in early human tumorigenesis. Nature.

[222] Bell D.W., Varley J.M., Szydlo T.E., Kang D.H., Wahrer D.C.R., Shannon K.E., Lubratovich M., Verselis S.J., Isselbacher K.J., Fraumeni J.F. (1999). Heterozygous germ line hCHK2 mutations in Li-Fraumeni syndrome. Science.

[223] Vahteristo P., Tamminen A., Karvinen P., Eerola H., Eklund C., Aaltonen L.A., Blomqvist C., Aittomaki K., Nevanlinna H. (2001). p53, CHK2, and CHK1 genes in Finnish families with Li-Fraumeni syndrome: Further evidence of CHK2 in inherited cancer predisposition. Cancer Res..

[224] Miller C.W., Ikezoe T., Krug U., Hofmann W.K., Tavor S., Vegesna V., Tsukasaki K., Takeuchi S., Koeffler H.P. (2002). Mutations of the CHK2 gene are found in some osteosarcomas, but are rare in breast, lung, and ovarian tumors. Genes Chromosomes Cancer.

[225] Nevanlinna H., Bartek J. (2006). The CHEK2 gene and inherited breast cancer susceptibility. Oncogene.

[226] Lee S.B., Kim S.H., Bell D.W., Wahrer D.C.R., Schiripo T.A., Jorczak M.M., Sgroi D.C., Garber J.E., Li F.P., Nichols K.E. (2001). Destabilization of CHK2 by a missense mutation associated with Li-Fraumeni syndrome. Cancer Res..

[227] Meijers-Heijboer H., Ouweland A.V.D., Klijn J., Wasielewski M., De Snoo A., Oldenburg R., Hollestelle A., Houben M., Crepin E., Van Veghel-Plandsoen M. (2002). Low-penetrance susceptibility to breast cancer due to CHEK2*1100delC in noncarriers of BRCA1 or BRCA2 mutations. Nat. Genet..

[228] Dong X., Wang L., Taniguchi K., Wang X., Cunningham J.M., McDonnell S.K., Qian C., Marks A.F., Slager S.L., Peterson B.J. (2003). Mutations in CHEK2 associated with prostate cancer risk. Am. J. Hum. Genet..

[229] Wu X., Dong X., Liu W., Chen J. (2006). Characterization of CHEK2 mutations in prostate cancer. Hum. Mutat..

[230] Cybulski C., Gorski B., Huzarski T., Masojc B., Mierzejewski M., Debniak T., Teodorczyk U., Byrski T., Gronwald J., Matyjasik J. (2004). CHEK2 is a multiorgan cancer susceptibility gene. Am. J. Hum. Genet..

[231] Chabalier-Taste C., Racca C., Dozier C., Larminat F. (2008). BRCA1 is regulated by Chk2 in response to spindle damage. Biochim. Et. Biophys. Acta.

[232] Choi W., Lee E.S. (2022). Therapeutic Targeting of DNA Damage Response in Cancer. Int. J. Mol. Sci..

[233] Gisselsson D. (2008). Classification of chromosome segregation errors in cancer. Chromosoma.

[234] Bai J., Wioland H., Advedissian T., Cuvelier F., Romet-Lemonne G., Echard A. (2020). Actin reduction by MsrB2 is a key component of the cytokinetic abscission checkpoint and prevents tetraploidy. Proc. Natl. Acad. Sci. USA.

[235] Norden C., Mendoza M., Dobbelaere J., Kotwaliwale C.V., Biggins S., Barral Y. (2006). The NoCut pathway links completion of cytokinesis to spindle midzone function to prevent chromosome breakage. Cell.

[236] Petsalaki E., Zachos G. (2016). Clks 1, 2 and 4 prevent chromatin breakage by regulating the Aurora B-dependent abscission checkpoint. Nat. Commun..

[237] Steigemann P., Wurzenberger C., Schmitz M.H.A., Held M., Guizetti J., Maar S., Gerlich D.W. (2009). Aurora B-mediated abscission checkpoint protects against tetraploidization. Cell.

[238] Honda R., Körner R., Nigg E.A. (2003). Exploring the functional inter- actions between Aurora B, INCENP, and survivin in mitosis. Mol. Biol. Cell.

[239] Adriaans I.E., Hooikaas P.J., Aher A., Vromans M.J.M., Van Es R.M., Grigoriev I., Akhmanova A., Lens S.M.A. (2020). MKLP2 Is a Motile Kinesin that Transports the Chromosomal Passenger Complex during Anaphase. Curr. Biol..

[240] Van der Horst A., Vromans M.J.M., Bouwman K., van der Waal M.S., Hadders M.A., Lens S.M.A. (2015). Inter-domain Cooperation in INCENP Promotes Aurora B Relocation from Centromeres to Microtubules. Cell Rep..

[241] Kim Y.B., Shin Y.J., Roy A., Kim J.H. (2015). The Role of the Pleckstrin Homology Domain-containing Protein CKIP-1 in Activation of p21-activated Kinase 1 (PAK1). J. Biol. Chem..

[242] Zhang D.G., Zhang J., Mao L.L., Wu J.X., Cao W.J., Zheng J.N., Pei D.S. (2015). p21-Activated kinase 5 affects cisplatin-induced apoptosis and proliferation in hepatocellular carcinoma cells. Tumour Biol..

[243] Kim S.T. (2005). Protein kinase CK2 interacts with Chk2 and phosphorylates Mre11 on serine 649. Biochem. Biophys. Res. Commun..

[244] Bjørling-Poulsen M., Siehler S., Wiesmüller L., Meek D., Niefind K., Issinger O.G. (2005). The ‘regulatory’ beta-subunit of protein kinase CK2 negatively influences p53-mediated allosteric effects on Chk2 activation. Oncogene.

[245] Kroonen J., Artesi M., Capraro V., Nguyen-Khac M.T., Willems M., Chakravarti A., Bours V., Robe P.A. (2012). Casein kinase 2 inhibition modulates the DNA damage response but fails to radiosensitize malignant glioma cells. Int. J. Oncol..

[246] Levine M.S., Holland A.J. (2018). The impact of mitotic errors on cell proliferation and tumorigenesis. Genes Dev..

[247] McKenzie C., D’Avino P.P. (2016). Investigating cytokinesis failure as a strategy in cancer therapy. Oncotarget.

[248] Zhang S., Zhu H. (2018). Cytokinesis and the Hippo Pathway: New Molecular Links Between Intimate Partners. Gastroenterology.

[249] Lens S.M.A., Medema R.H. (2019). Cytokinesis defects and cancer. Nat. Rev. Cancer.

