# Supplementary figures and images for: Minor Kinases with Major Roles in Cytokinesis Regulation
Source: Cells. 2022 Nov 17;11(22):3639. doi: 10.3390/cells11223639 (PMC9688779; doi:10.3390/cells11223639)

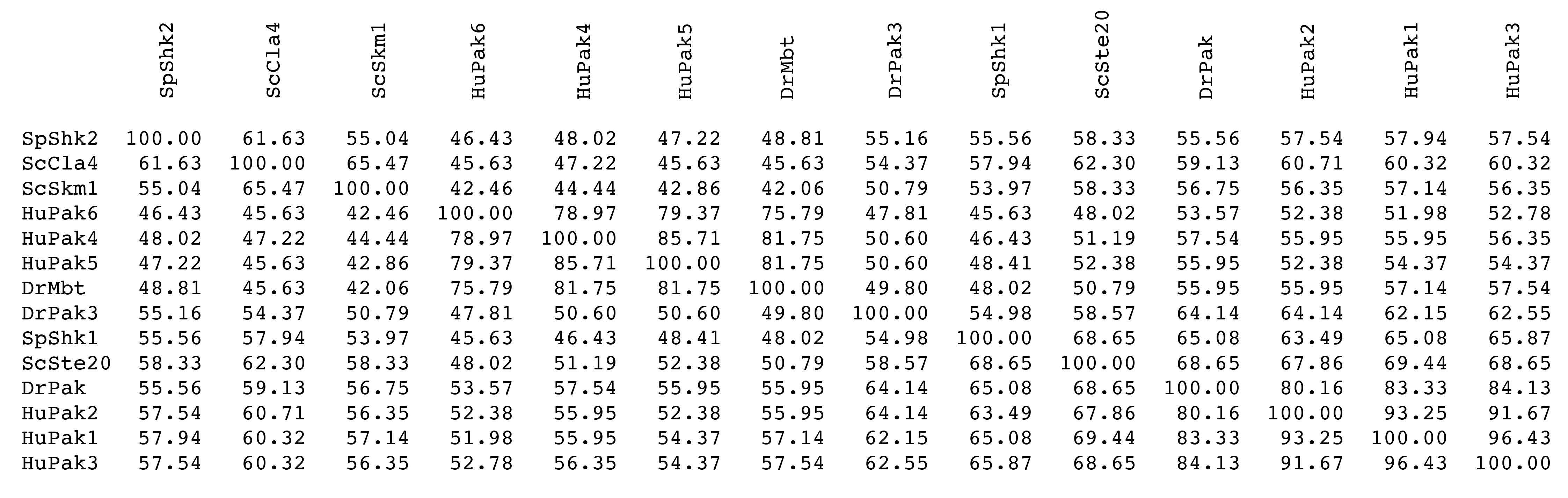

Supplement: Supplementary file 1 [file cells-11-03639-s001.zip › cells-1986307-supplementary-Figure S1.tif]
